# Spectral Shape Recovery and Analysis Via Data-driven Connections

**DOI:** 10.1007/s11263-021-01492-6

**Published:** 2021-07-22

**Authors:** Riccardo Marin, Arianna Rampini, Umberto Castellani, Emanuele Rodolà, Maks Ovsjanikov, Simone Melzi

**Affiliations:** 1 Sapienza University of Rome, Rome, Italy; 2 University of Verona, Verona, Italy; 3 LIX, Ecole Polytechnique, IP Paris, France

**Keywords:** Shape from spectrum, Spectral geometry, Shape analysis, Representation learning, Geometry processing

## Abstract

We introduce a novel learning-based method to recover shapes from their Laplacian spectra, based on establishing and exploring connections in a learned latent space. The core of our approach consists in a cycle-consistent module that maps between a learned latent space and sequences of eigenvalues. This module provides an efficient and effective link between the shape geometry, encoded in a latent vector, and its Laplacian spectrum. Our proposed data-driven approach replaces the need for ad-hoc regularizers required by prior methods, while providing more accurate results at a fraction of the computational cost. Moreover, these latent space connections enable novel applications for both analyzing and controlling the spectral properties of deformable shapes, especially in the context of a shape collection. Our learning model and the associated analysis apply without modifications across different dimensions (2D and 3D shapes alike), representations (meshes, contours and point clouds), nature of the latent space (generated by an auto-encoder or a parametric model), as well as across different shape classes, and admits arbitrary resolution of the input spectrum without affecting complexity. The increased flexibility allows us to address notoriously difficult tasks in 3D vision and geometry processing within a unified framework, including shape generation from spectrum, latent space exploration and analysis, mesh super-resolution, shape exploration, style transfer, spectrum estimation for point clouds, segmentation transfer and non-rigid shape matching.

**Supplementary Information:**

The online version supplementary material available at 10.1007/s11263-021-01492-6.

## Introduction

Constructing compact encodings of geometric shapes lies at the heart of 2D and 3D Computer Vision. While earlier approaches have concentrated on handcrafted representations, with the advent of geometric deep learning (Bronstein et al. 2017; Masci et al. 2016), data-driven *learned* feature encodings have gained prominence. A desirable property in many applications, such as shape exploration and synthesis, is to be able to recover the shape from its (latent) encoding or to control the object deformations in a parametric fashion. Various data-driven parametric models (Loper et al. 2015; Zuffi et al. 2017; Romero et al. 2017; Pavlakos et al. 2019) and auto-encoder architectures have been designed to solve this problem (Achlioptas et al. 2018; Litany et al. 2018; Mo et al. 2019; Gao et al. 2019). Despite significant progress in this area, the structure of the latent vectors is arduous to control. For example, the dimensions of the latent vectors typically lack a canonical ordering, while invariance to various geometric deformations is often only learned by data augmentation or complex constraints on the intermediate features.

**Fig. 1:**
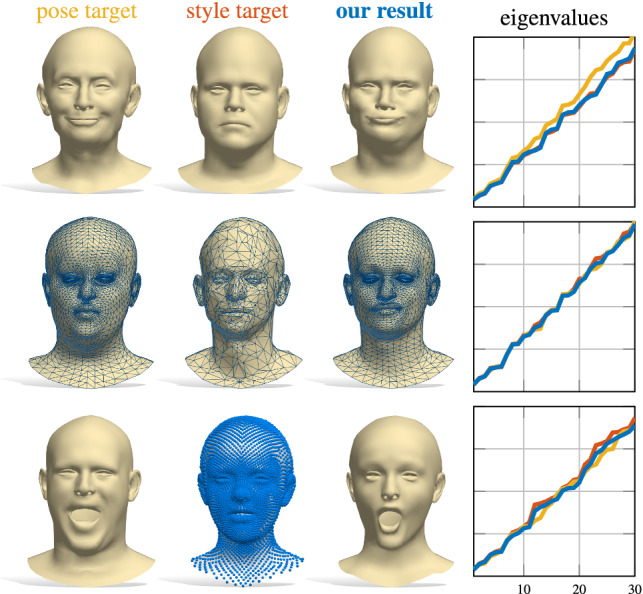
Our spectral reconstruction enables correspondence-free style transfer. Given pose and style “donors” (left and middle columns respectively), we synthesize a new shape with the pose of the former and the style of the latter. The generation is driven by a learning-based eigenvalues alignment (rightmost plots). Our approach handles different resolutions (middle row) and representations (bottom row; the surface underlying the point cloud is for visualization purposes only)

At the same time, a classical approach in spectral geometry is to encode a shape using the set of increasingly ordered eigenvalues (spectrum) of its Laplacian operator. This representation is useful since: (1) it does not require any training, (2) it can be computed on various data representations, such as point clouds or meshes, regardless of sampling density, (3) it enjoys well-known theoretical properties such as a natural ordering of its elements and invariance to isometries, and (4) as shown recently (Cosmo et al. 2019; Rampini et al. 2019), alignment of eigenvalues often promotes near-isometries, which is useful in multiple tasks such as non-rigid shape retrieval and matching problems.

Unfortunately, although encoding shapes via their Laplacian spectra can be straightforward (at least for meshes), the inverse problem of recovering the shape is very difficult. Indeed, it is well-known that certain pairs of non-isometric shapes can have the same spectrum, or in other words “one cannot hear the shape of a drum” (Gordon et al. 1992). At the same time, recent evidence suggests that such cases are pathological and that *in practice* it might be possible to recover a shape from its spectrum (Cosmo et al. 2019). Nevertheless, existing approaches (Cosmo et al. 2019), while able to deform a shape into another with a given spectrum, can produce highly unrealistic shapes with strong artifacts failing in a large number of cases.

**Fig. 2:**
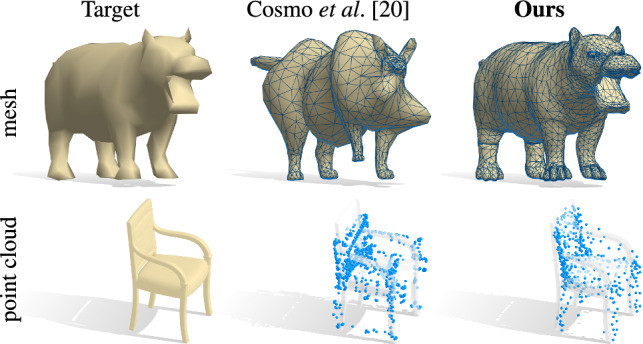
Comparison in estimating a shape from its Laplacian spectrum between the state-of-the-art method (Cosmo et al. 2019) (middle) and ours (right) for a mesh and a point cloud. The shapes recovered by our method are significantly closer to the target

In this paper, we combine the strengths of data-driven latent representations with those of spectral methods. Our key idea is to construct a connection between the space of Laplacian spectra and a learned latent space. This connection allows us to synthesize shapes from either their learned latent codes or their Laplacian eigenvalues, and further provides us with a way to explore the latent space by an intuitive manipulation of eigenvalues. Moreover, we demonstrate that this process endows the latent space with certain desirable properties that are missing in standard auto-encoder architectures. Our shape-from-spectrum solution is very efficient since it requires a single pass through a trained network, unlike expensive iterative optimization methods with ad-hoc regularizers (Cosmo et al. 2019; Rampini et al. 2019). Furthermore, our trainable module acts as a proxy to differentiable eigendecomposition, while encouraging geometric consistency within the network. Overall, our key **contributions** can be summarized as follows:

- We propose the first learning-based model to robustly recover shapes from Laplacian spectra *in a single pass*;
- For the first time, we provide a bidirectional connection between learned latent spaces and spectral geometric properties of 3D shapes, giving rise to new tools for the analysis of geometric data;
- Our model is *general*, in that it applies with no modifications to different classes even across different geometric representations and dimensions, and generalizes to representations not available at training time;
- Our connections can be applied to different kinds of latent representation, such as the ones provided by auto-encoders or from parametric models;
- We showcase our approach in multiple applications (e.g., Fig. 1), and show significant improvement over the state of the art; see Fig. 2 for an example.

## Related Work

Spectral quantities and in particular the eigenvalues of the Laplace-Beltrami operator provide an informative summary of the intrinsic geometry. For example, closed-form estimates and analytical bounds for surface area, genus and curvature in terms of the Laplacian eigenvalues have been obtained (Chavel 1984). Given these properties, spectral shape analysis has been exploited in many computer vision and computer graphics tasks such as shape retrieval (Reuter et al. 2005), description and matching (Sun et al. 2009; Aubry et al. 2011; Bronstein et al. 2011; Ovsjanikov et al. 2012), mesh segmentation (Reuter 2010), sampling (Öztireli et al. 2010) and compression (Karni and Gotsman 2000) among many others. Typically, the intrinsic properties of the shape are computed from its explicit representation and are used to encode compact geometric features invariant to isometric deformations.

Recently, several works have started to address the inverse problem: namely, recovering an extrinsic embedding from the intrinsic encoding (Boscaini et al. 2015; Cosmo et al. 2019). This is closely related to the fundamental theoretical question of “hearing the shape of the drum” (Kac 1966; Gordon et al. 1992). Although counter-examples have been proposed to show that in certain scenarios multiple shapes might have the same spectrum, there is recent work that proposes effective practical solutions to this problem. In Boscaini et al. (2015) the shape-from-operator method was proposed, aiming at obtaining the extrinsic shape from a Laplacian matrix where the 3D reconstruction was recovered after the estimation of the Riemannian metric in terms of edge lengths. In Corman et al. (2017) the intrinsic and extrinsic relations of geometric objects have been extensively defined and evaluated from both theoretical and practical aspects. The authors revised the framework of functional shape differences (Rustamov et al. 2013) to account for extrinsic structure, extending the reconstruction task to non-isometric shapes and models obtained from physical simulation and animation. Several works have also been proposed to recover shapes purely from Laplacian *eigenvalues* (Chu and Golub 2005; Aasen et al. 2013; Panine and Kempf 2016) or with mild additional information such as excitation amplitude in the case of musical key design (Bharaj et al. 2015). Most closely related to ours in this area is the recent *isopectralization* approach introduced in Cosmo et al. (2019), that aims directly to estimate the 3D shape from the spectrum. This approach works well in the vicinity of a good solution but is both computationally expensive and, as we show below, can quickly produce unrealistic instances, failing in a large number of cases in 3D, as shown in Fig. 2 for two examples.

In this paper we contribute to this line of work, and propose to replace the heuristics used in previous methods, such as Cosmo et al. (2019), with a purely data-driven approach for the first time. Our key idea is to design a deep neural network, that both constraints the space of solutions based on the set of shapes given at training, and at the same time, allows us to solve the isospectralization problem with a *single forward pass*, thus avoiding expensive and error-prone optimization.

We note that a related idea has been recently proposed in Huang et al. (2019) via the so-called OperatorNet architecture. However, that work is based on shape difference operators (Rustamov et al. 2013) and as such requires a fixed source shape and functional maps to each shape in the dataset to properly synthesize a shape. Our approach is based on Laplacian eigenvalues alone, and thus is completely correspondence-free.

Our approach also builds upon the recent work on learning generative shape models. A range of techniques have been proposed using volumetric representations (Wu et al. 2016), parametric models (Loper et al. 2015; Pavlakos et al. 2019; Zuffi et al. 2017), point cloud auto-encoders (Aumentado-Armstrong et al. 2019; Achlioptas et al. 2018), generative models based on meshes and implicit functions (Sinha et al. 2017; Groueix et al. 2018; Litany et al. 2018; Kostrikov et al. 2018; Chen and Zhang 2019), and part structures (Li et al. 2017; Mo et al. 2019; Gao et al. 2019; Wu et al. 2019), among many others. Although generative models, and auto-encoders in particular, have shown impressive performance, the structure of the latent space is typically difficult to control or analyze directly. To address this problem, some methods proposed a disentanglement of the latent space (Wu et al. 2019; Aumentado-Armstrong et al. 2019) to split it in more semantic regions. Perhaps most closely related to ours in this domain, is the work in Aumentado-Armstrong et al. (2019), where the shape spectrum is used to promote disentanglement of the latent space into intrinsic and extrinsic components, that can be controlled separately. Nevertheless, the resulting network does not allow to synthesize shapes from their spectra.

Extending the studies of these approaches, our work provides the first way to connect the learned latent space to the spectral one, thus inheriting the benefits and providing the versatility of moving across the two representations. This allows our network to synthesize shapes from their spectra, and also to relate shapes with very different input structure (*e*.*g*., meshes and point clouds) across a vastness of sampling densities, enabling several novel applications.

This paper is an extended version of the work presented in Marin et al. (2020). Compared to the former version, our contribution is as follows: (i) We investigate different types of latent space, including those generated by an auto-encoder model as well as parametric spaces associated with morphable models, and study different parametrizations thereof; (ii) we include human bodies among the classes of analyzed shapes; (iii) we further develop the tools provided by our model for a meaningful exploration of the latent space, showing how the spectral prior contributes to the interpretability of latent codes, and enabling the disentanglement of intrinsic and extrinsic geometry as a novel application (Sect. 6); (iv) we introduce non-rigid matching as a new application of the shape-from-spectrum paradigm (Sect. 7).

## Background

We model shapes as connected 2-dimensional Riemannian manifolds $\mathcal {X}$ embedded in $\mathbb {R}^3$, possibly with boundary $\partial \mathcal {X}$, equipped with the standard metric. On each shape $\mathcal {X}$ we consider its positive semi-definite Laplace-Beltrami operator $\varDelta _\mathcal {X}$, generalizing the classical notion of Laplacian from the Euclidean setting to curved surfaces.

**Laplacian spectrum.** $\varDelta _\mathcal {X}$ admits an eigendecomposition

1 $$\begin{aligned} \varDelta _\mathcal {X}\phi _i(x) = \lambda _i \phi _i(x) \quad ~~&x\in \mathrm {int}(\mathcal {X}) \end{aligned}$$

2 $$\begin{aligned} \langle \nabla \phi _i(x), \hat{n} (x) \rangle = 0 \quad ~~&x\in \partial \mathcal {X} \end{aligned}$$

into eigenvalues $\{\lambda _i\}$ and associated eigenfunctions $\{\phi _i\}$^1^.

The Laplacian eigenvalues of $\mathcal {X}$ (its *spectrum*) form a discrete set, which is canonically ordered into a non-decreasing sequence

3 $$\begin{aligned} \mathrm {Spec}(\mathcal {X}) := \{0=\lambda _0<\lambda _1\le \lambda _2\le \cdots \}\,. \end{aligned}$$

In the special case where $\mathcal {X}$ is an interval in $\mathbb {R}$, the eigenvalues $\lambda _i$ correspond to the (squares of) oscillation frequencies of Fourier basis functions $\phi _i$. This provides us with a connection to classical Fourier analysis, and with a natural notion of hierarchy induced by the ordering of the eigenvalues. In the light of this analogy, in practice, one is usually interested in a limited bandwidth consisting of the first *k* eigenvalues; typical values in geometry processing applications range from $k=30$ to 100.

Furthermore, the spectrum is *isometry-invariant*, *i*.*e*., it does not change with deformations of the shape that preserve geodesic distances (*e*.*g*., changes in pose).

**Fig. 3:**
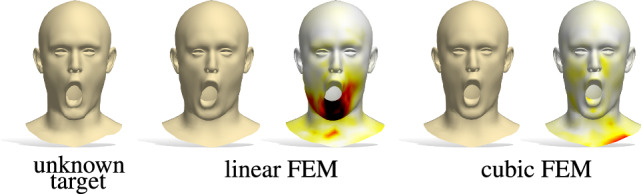
Reconstruction examples of our shape-from-spectrum pipeline. We show the results obtained with two different inputs: the eigenvalues of the Laplacian discretized with linear FEM, and those of the cubic FEM discretization. The heatmap encodes point-wise reconstruction error, growing from white to dark red

**Discretization.** In the discrete setting, we represent shapes as triangle meshes $X=(V,T)$ with *n* vertices *V* and *m* triangular faces *T*; depending on the application, we will also consider unorganized point clouds. Vertex coordinates in both cases are represented by a matrix $\mathbf {X}\in \mathbb {R}^{n\times 3}$.

The Laplace-Beltrami operator $\varDelta _\mathcal {X}$ is discretized as a $n\times n$ matrix via the finite element method (FEM) (Ciarlet 2002). In the simplest setting (*i*.*e*., linear finite elements), this discretization corresponds to the cotangent Laplacian (Pinkall and Polthier 1993); however, in this paper we consider both *quadratic* FEM and *cubic* FEM (see, *e*.*g*., (Reuter 2010, Sec. 4.1) for a clear treatment). These yield a more accurate discretization as shown in Fig. 3 and evaluated in Table 2. Differently from Cosmo et al. (2019), Rampini et al. (2019), this comes at virtually no additional cost for our pipeline, as we will show. On point clouds, $\varDelta _\mathcal {X}$ can be discretized using the approach described in Clarenz et al. (2004), Boscaini et al. (2016).

## Method

Our main contribution is a deep learning model for recovering shapes from Laplacian eigenvalues. Our model operates in an end-to-end fashion: given a spectrum as input, it directly yields a shape with a single forward pass, thus avoiding expensive test-time optimization.

**Motivation.** Our rationale lies in the observation that shape semantics can be learned from the data, rather than by relying upon the definition of ad-hoc regularizers (Cosmo et al. 2019), often resulting in unrealistic reconstructions. For example, a sheet of paper can be *isometrically* crumpled or folded into an airplane (see inset figure). Since both embeddings have exactly the same eigenvalues, the desired reconstruction must be imposed as a prior. By taking a data-driven approach, we make our method aware of the “space of realistic shapes”, yielding both a dramatic improvement in accuracy and efficiency, and enabling new interactive applications.

### Latent Space Connections for Auto-encoders

**Fig. 4:**
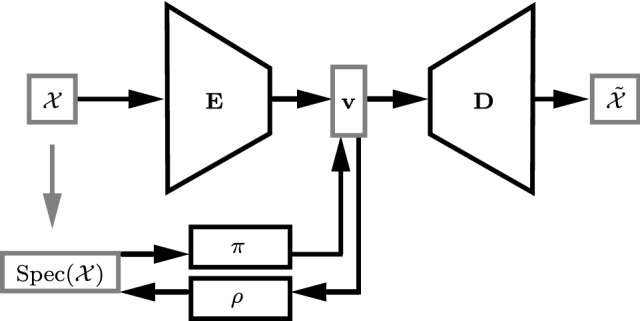
Our network model. The input shape $\mathcal {X}$ and its Laplacian spectrum $\mathrm {Spec}(\mathcal {X})$ are passed, respectively, through an AE enforcing $\mathcal {X}\approx \tilde{\mathcal {X}}$, and an invertible module $(\pi ,\rho )$ mapping the eigenvalue sequence to a latent vector $\mathbf {v}$. The two branches are trained simultaneously, forcing $\mathbf {v}$ to be updated accordingly. The trained model allows to recover the shape purely from its eigenvalues via the composition $D(\pi (\mathrm {Spec}(\mathcal {X})))\approx \mathcal {X}$

Our first key contribution is to construct an auto-encoder (AE) neural network architecture, augmented by explicitly modeling the connections between the latent space of the AE and the Laplacian spectrum of the input shape; see Fig. 4 for an illustration of this learning model.

Loosely speaking, our approach can be seen as implementing a coupling between two latent spaces: a learned one that operates on the shape embedding $\mathcal {X}$, and the one provided by the eigenvalues $\mathrm {Spec}(\mathcal {X})$. In the former case, the encoder *E* is trainable, whereas the mapping $\mathcal {X}\rightarrow \mathrm {Spec}(\mathcal {X})$ is provided via the eigen-decomposition and fixed a priori. Further, we introduce the two coupling mappings $\pi , \rho$, trained with a bidirectional loss, to both enable communication across the latent spaces and to tune the learned space by endowing it with structure contained in $\mathrm {Spec}(\mathcal {X})$.

We phrase our overall training loss as follows:

4 $$\begin{aligned} \ell&= \ell _\mathcal {X}+ \alpha \ell _\lambda \,,\quad \mathrm {with} \end{aligned}$$

5 $$\begin{aligned} \ell _\mathcal {X}&= \frac{1}{n}\Vert D(E(\mathbf {X})) - \mathbf {X} \Vert _F^2 \end{aligned}$$

6 $$\begin{aligned} \ell _\lambda&= \frac{1}{k}(\Vert \pi ({\varvec{\lambda }}) - E(\mathbf {X}) \Vert _2^2 + \Vert \rho (E(\mathbf {X})) - {\varvec{\lambda }} \Vert _2^2) \end{aligned}$$

where ${\varvec{\lambda }}$ is a vector containing the first *k* (positive) eigenvalues in $\mathrm {Spec}(\mathcal {X})$, $\mathbf {X}$ is the matrix of point coordinates, *E* is the encoder, *D* is the decoder (Fig. 4), $\Vert \cdot \Vert _F$ denotes the Frobenius norm, and $\alpha =10^{-4}$ controls the relative strengths of the reconstruction loss $\ell _\mathcal {X}$ and the spectral term $\ell _\lambda$. The blocks *D*, *E*, $\pi$, and $\rho$ are learnable and parametrized by neural networks (see the supplementary material for the implementation details). Eq. (6) enforces $\rho \approx \pi ^{-1}$; in other words, $\pi$ and $\rho$ form a translation block between the latent vector and the spectral encoding of the shape.

At test time, we recover a shape from a given spectrum $\mathrm {Spec}(\mathcal {X})$ simply via the composition $D(\pi (\mathrm {Spec}(\mathcal {X})))$ (Sect. 5). For additional applications we refer to Sect. 8.

**Shape representation.** We consider two different settings: triangle meshes in point-to-point correspondence *at training time* (typical in graphics and geometry processing), and unorganized point clouds *without* a consistent vertex labeling (typical in 3D computer vision).

**Auto-encoder architecture.** Our model can be built with potentially any AE. In our applications we chose relatively simple ones to deal with meshes and unorganized point clouds, although more powerful generative methods would be equally possible.

***Remark.*** Our architecture takes $\mathrm {Spec}(\mathcal {X})$ as an input, *i*.*e*., the eigenvalues are *not* computed at training time. By learning an invertible mapping to the latent space, we avoid expensive backpropagation steps through the spectral decomposition of the Laplacian $\varDelta _\mathcal {X}$. In this sense, the mapping $\rho$ acts as an efficient proxy to differentiable eigendecomposition, which we exploit in several applications below.

Since eigenvalue computation is only incurred as an offline cost, it can be performed with arbitrary accuracy (we use cubic FEM, see Fig. 3 and Table 2) without sacrificing efficiency. We refer to the supplementary material for details about the architecture, both in the case of meshes and point clouds. In all our experiments, we set the latent space dimension *k* equal to the number of eigenvalues $\mathrm {Spec}(\mathcal {X})$, specifically $k =30$. In Sect. 5.2, we compare the results for different choices of *k*.

### Latent Space Connections for Parametric Models

**Fig. 5:**
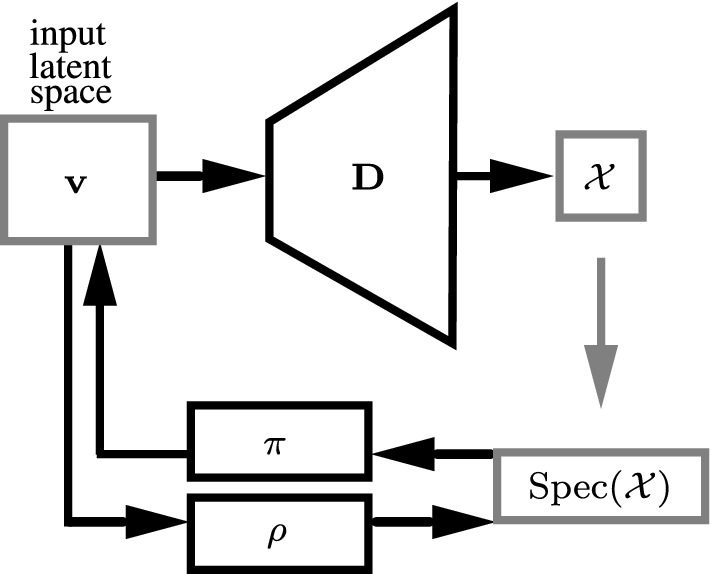
In the morphable model setting, the latent space (*i*.*e*., the space of deformation parameters) is given together with its decoder $\mathbf {D}$; both are fixed at training time

Our second key idea is to connect the Laplacian spectrum with the space of parameters of a given morphable model. We illustrate this construction in Fig. 5. This approach is similar to the previous one, with two important differences: 1) there is no encoder involved in the loop; 2) the latent space is also given as input, i.e., it is not learned during training. As before, we establish the connection between the two given representations by training the networks $\pi$ and $\rho$ with a bidirectional loss, which is similar to Eq. (6):

7 $$\begin{aligned} {\widehat{\ell }_\lambda } {=} {\frac{1}{k}(\Vert \pi ({\varvec{\lambda }}) - \mathbf {v} \Vert _2^2 + \Vert \rho (\mathbf {v}) - {\varvec{\lambda }} \Vert _2^2)\,,} \end{aligned}$$

where all the symbols have the same meaning as in the previous losses. The equation above can be obtained from Eq. (6) by replacing $E(\mathbf {X})$ with $\mathbf {v}$, and replacing the learned encoded representation with a fixed one.

**Parametric models.** We consider two different parametric models, namely, the seminal model SMPL (Loper et al. 2015), and its updated version SMPL-X (Pavlakos et al. 2019). Despite dealing with similar data (human bodies), these two models have very different parametric spaces.

## Results and analysis

In this section we report the results on our core application of shape from spectrum recovery, together with an analysis of the various parameters and timing.

### Shape-from-spectrum recovery

To evaluate our method, we trained our model on 1853 3D shapes from the CoMA dataset (Ranjan et al. 2018) of human faces; 100 shapes of an unseen subject are used for the test set. We repeated this test at four different mesh resolutions: $\sim$4K (full resolution), 1K, 500, and 200 vertices respectively. For each resolution, we independently compute the Laplacian spectrum and use these spectra to recover the shape.

**Table 1:** Shape-from-spectrum reconstruction comparisons with NN (nearest neighbors between spectra) and the state of the art (Cosmo et al. 2019); we report an average error over 100 shapes of an unseen subject from COMA (Ranjan et al. 2018). Best results are obtained with our full pipeline

	full res	1000	500	200
**Ours**	$\mathbf {1.61}$	$\mathbf {1.62}$	$\mathbf {1.71}$	$\mathbf {2.13}$
Ours without $\rho$	1.89	1.82	2.06	2.42
NN	4.45	4.63	4.01	2.65
Cosmo et al. (2019)	−	16.4	7.11	4.08

‘−’ denotes out of memory; all errors must be rescaled by $10^{-5}$

**Comparison.** We compared our method in terms of reconstruction accuracy to the state-of-the-art isospectralization method of Cosmo et al. (2019), as well as to a nearest-neighbors baseline, consisting in picking the shape of the training set with the closest spectrum to the target one. In addition, we trained two separate architectures (with and without the $\rho$ block) and compared them. The test without this network component is an ablation study we carry out to validate the importance of the *invertible* module connecting the spectral encoding to the learned latent codes.

**Fig. 6:**
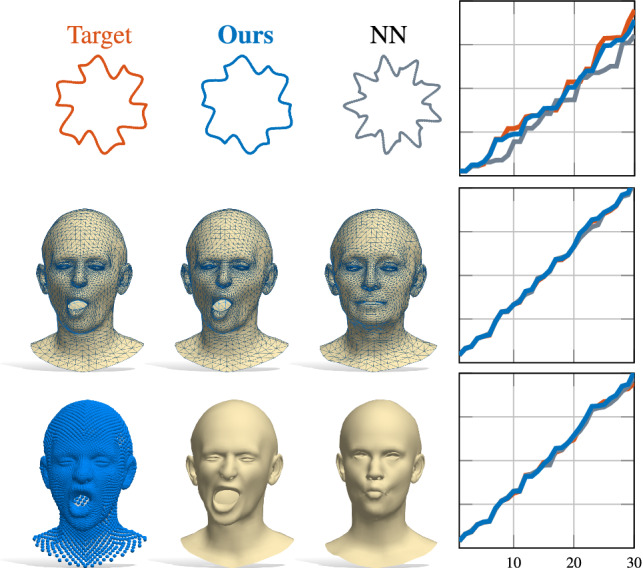
Shape reconstruction from eigenvalues using our approach on different representations (i.e. 2D contours, 3D meshes and point clouds). The eigenvalues of the shapes on the left are fed into our network, which outputs the shapes in the middle. For each representation, the eigenvalues are computed on the appropriate Laplacian discretization as per Sect. 3. The NN column shows the nearest-neighbor solution sought in the training set

The quantitative results are reported in Table 1 as the mean squared error between the reconstructed shape and the ground-truth. Figures 2 and 6 further show qualitative comparisons with the different baselines on different shape representations. In Fig. 6, for the sake of illustration and similarly to Cosmo et al. (2019), Rampini et al. (2019), we also include 2D contours discretized as regular cycle graphs. As the results suggest, the $\rho$ block both contributes to reduce the reconstruction error, and to enable novel applications (we explore them in depth in Sec. 8). Our method achieves a significant improvement over nearest neighbors in terms of accuracy, and an order of magnitude improvement over isospectralization Cosmo et al. (2019). Also, the latter approach consists in an expensive optimization which requires hours to run, while our method is instantaneous at test time.

**Fig. 7:**
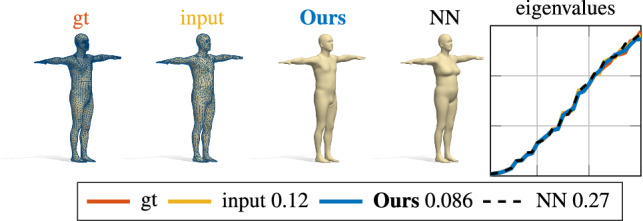
Shape-from-spectrum reconstruction of a test shape from the SURREAL dataset (Varol et al. 2017). The subject was not seen at training time, and has a different discretization than the training shapes

**Fig. 8:**
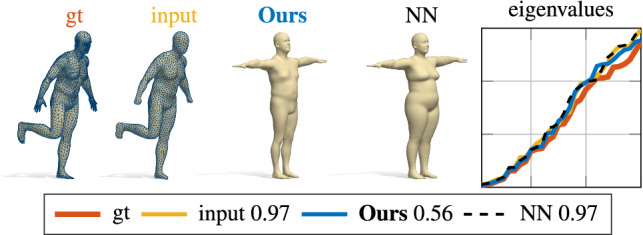
Shape-from-spectrum reconstruction of a test shape from the FAUST dataset (Bogo et al. 2014). The input mesh has different connectivity as well as different pose from the shapes in the training set

We perform further experiments on the human bodies category, by training our model on a set of 3014 shapes (in T-Pose) from the SURREAL dataset Varol et al. (2017). The quantitative evaluation is reported on different test sets in the fifth column of Table 2, and qualitatively in Figures 7 and 8. In the qualitative examples, the shapes have been remeshed to have a different connectivity from the ones seen at training time. The numbers reported in the legend encode the relative error $\sum _i (\lambda _i^\mathrm {gt} - \lambda _i)^2/(\lambda _i^\mathrm {gt})^2$, where $\lambda _i^\mathrm {gt}$ are the ground-truth eigenvalues, while $\lambda _i$ are the eigenvalues of the shapes as labeled in the figures (the smaller, the better).

**Fig. 9:**
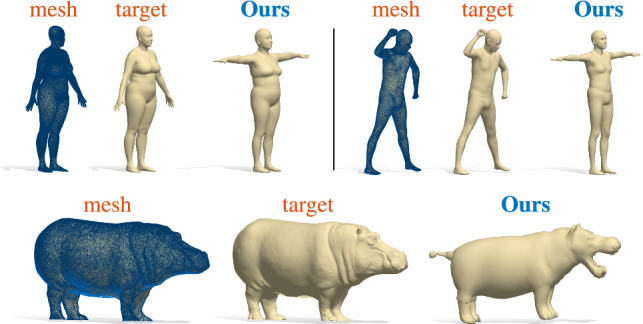
Shape-from-spectrum reconstruction of test shapes outside the training distribution. First row: two human shapes from SHREC19; second row: a hippo from SHREC20. From left to right of each block, the target shape with its overlaid mesh, the target surface, and the output of our model

Finally, in Figure 9, we test our model on shapes that are outside the training distribution. In the first row, two target human shapes selected from the SHREC19 benchmark Melzi et al. (2019). In the second row, an example on animals for a shape from SHREC20 Dyke et al. (2020). Even if the input geometry is far from the training distribution, our model is able to provide meaningful results that respect the main semantic features of the target shape. For example, with the hippo shape in the bottom row, several features of the target shape are missing in our result, but we are able to retrieve the global geometry and the correct class among the ones present in the training set. We remark that these shapes are very challenging, since they come from different datasets, represent different subjects, different poses, and are discretized with completely different meshes.

**Table 2:** Quantitative ablation study on different test sets (one per row), with different variants of our model (one per column)

	$O_{10}$	$O_{15}$	$O^1_{30}$	$O^2_{30}$	$O_{30}$	$O_{60}$	$P_{30}$	$NN_{30}$	$S_{10}$	$SX_{15}$	$SX_{30}$	$SX_{60}$	*VAE*
SURREAL	20.34	20.12	2.92	2.88	1.68	1.52	2.38	15.96	27.24	42.02	41.08	44.31	1.34
SURREAL rem	20.64	20.17	13.85	7.13	2.32	2.46	2.84	17.90	25.09	42.95	41.92	46.30	1.97
SURREAL uni	230	180	340	280	200	270	370	240	480	320	240	170	205
FAUST	397	442	535	509	385	364	373	369	450	197	200	200	377
FAUST rem	390	434	533	434	378	362	364	365	439	198	200	200	371
FAUST uni	787	539	517	419	445	444	665	915	263	198	200	200	383

$O_{k}$ are based on an AE for meshes, $P_{k}$ adopts the AE for pointcloud, *VAE* exploits a variational AE, $S_{k}$ and $SX_{k}$ use the latent space from the parametric models SMPL and SMPL-X respectively. Parameter *k* is the dimension of the latent space and the number of strictly positive eigenvalues. The indices 1 and 2 denote the linear and quadratic FEM respectively, otherwise we use the cubic FEM. *NN* is the baseline that returns the shape in the training set that has the most similar spectrum to the input one. All the results are scaled by $10^{5}$ for easier reading

### Ablation Study

We conducted an in-depth ablation study on the human body category, for which we can easily compare across the different latent spaces introduced in the previous Section. In Table 2 we compare different variants of our learning models:

- $O_{k}$ is our AE-based model (Fig. 4) for meshes;
- $P_{k}$ is the same as $O_{k}$, but for point clouds;
- $\textit{VAE}$ is a probabilistic variant of our AE-based model, obtained by replacing the deterministic AE with a variational autoencoder with the same architecture;
- $S_{k}$ and $SX_{k}$ are based on the parametric models SMPL and SMPL-X, respectively (Fig. 5);
- *NN* is the baseline; for every input spectrum, it outputs the training shape with the most similar spectrum (we use the Euclidean distance).

Parameter *k* denotes the dimension of the latent space (equal to the number of eigenvalues different from 0). The superscript indices 1 and 2 denote whether the eigenvalues are computed with a linear or quadratic FEM, respectively; in all the other cases, we use cubic FEM. The main difference between the two morphable models is in the dimension of the parametric space: 10 for SMPL and 400 for SMPL-X. For this reason, we can only select $k=10$ for SMPL ($S_{10}$), and different values of *k* for SMPL-X ($SX_{15}, SX_{30}, SX_{60}$). We report the performance of these models in the last 4 columns of Table 2, and refer to the supplementary materials for further details. These comparisons serve to motivate our choice of taking a fully data-driven approach over more straightforward, parametric alternatives. The parametric space provided by the morphable models is given, and not learned, together with the maps $\rho$ and $\pi$. Moreover, in this case, the decoding consists of a linear operation in contrast to the non-linear decoder of our network. The lower performance of the parametric model-based solutions show that non-linear operations achieve better results and that it is preferable to learn the latent space together with the bi-directional linkage to the space of spectra.

While the training set is fixed, we consider different test sets with an increasing level of difficulty:

- SURREAL: 755 shapes from the SURREAL dataset with the same pose and connectivity as the training shapes, but unseen subject;
- SURREAL rem: remeshed version of the former, ranging from $25\%$ to $70\%$ of the original number of vertices (see Fig. 7 for an example);
- SURREAL uni: remeshed version with uniform density, causing loss of detail for several thin subparts (see the top left shape of Fig. 16 for an example).

In these test sets, all the shapes are in the same pose and the ground truth is available. We measure the mean squared error between the 3D coordinates of the ground truth vertices and those of the shape recovered from the spectrum.

**Number of eigenvalues.** The comparison in Table 2 is done with different values of $k = 10, 15,30,60$. This parameter has a direct effect on reconstruction accuracy, since increasing this number brings more high-frequency detail into the representation. At the same time, the variations in the high frequencies are more unstable and so less easy to model in the data driven approach. The choice $k=30$ empirically leads to more stable results, confirming previous work in spectral geometry processing (Cosmo et al. 2019; Rampini et al. 2019; Roufosse et al. 2019). We use $k=30$ in all the following experiments, and report additional results for different *k* in the supplementary material.

**Robustness to different connectivity.** Our method is robust even under significant remeshing (uni), as shown in Figures 7, 8, and  16 (top left). This strong variation in the discretization still causes geometric distortion, which motivates the larger errors in the third row of Table 2. Despite the quantitative results indicate larger numerical error, however, qualitatively our approach still provides acceptable results in this challenging setting as shown in the Figures.

**FEM order.** We further compare the performance of our method using FEM of different orders for the computation of the eigenvalues: linear $O^1_{30}$, quadratic $O^2_{30}$ and cubic $O_{30}$. The results in Table 2 confirm that higher order FEM leads to more accurate results on all the test sets.

**Autoencoder architectures.** As mentioned in the previous section, we can build our model on top of any autoencoder. In Table 2 we compare two different architectures: one for meshes ($O_{30}$) and one for unorganized point clouds ($P_{30}$). The main difference is that $P_{30}$ exploits PointNet (Qi et al. 2017) as an encoder, and does not use any connectivity information between the vertices. More details about the two architectures are reported in the supplementary materials. The mesh-based architecture $O_{30}$ outperforms $P_{30}$, as expected from the additional information brought in by mesh connectivity. At the same time, $P_{30}$ outperforms the baseline $NN_{30}$, as well as the mesh-based architectures with fewer eigenvalues $O_{10}$, $O_{15}$ and the lower order FEM models $O_{30}^1$, $O_{30}^2$.

Finally, we test a probabilistic version of our pipeline involving a basic variational autoencoder (VAE). The resulting model is easily comparable with the other architectures proposed in the paper. Our VAE shares the same architecture of the AE with latent space of size $k=30$, and we used cubic FEM for the computation of eigenvalues of the training set. In this case, the training loss becomes:

8 $$\begin{aligned} \ell = \ell _\mathcal {X}+ \alpha \ell _\lambda + \beta \ell _{KL}\,, \end{aligned}$$

where $\ell _{KL} = D_{KL} (Q(\mathbf {v}|\mathcal {X}) | P(\mathbf {v}))$ is the Kullback-Leibler divergence to promote a Gaussian distribution in the latent space, with $Q(\mathbf {v}|\mathcal {X})$ being the posterior distribution given an input shape $\mathcal {X}$, and $P(\mathbf {v})$ being the Gaussian prior. In the last column of Table 2, we report the results obtained with this model. We note a slight improvement of the reconstruction error on all the considered benchmarks. This result suggests that more complex probabilistic generative models (e.g. exploiting the mesh hierarchy) and additional refinement of our method for applications requiring a high level of accuracy are promising directions for further investigation.

**Generalization to different data.** Finally, we tested on the FAUST dataset (Bogo et al. 2014), which is a data distribution outside of the training data SURREAL. Also in this case, we generated three different test sets: FAUST, FAUST rem and FAUST uni (last 3 rows of Table 2). These shapes are registrations of real human bodies, and are far from the ones seen at training time in terms of pose and subject (see Fig. 8 for an example). The task here is to evaluate the generalization capabilities of our model; given as input the eigenvalues of a FAUST shape in arbitrary pose, we aim to recover the FAUST shape in T-pose by using our model trained on SURREAL data. For the evaluation, we are given the ground-truth correspondence between the shapes from FAUST and SURREAL, and use it to compute the metric distortion between the two. This different error measure motivates the different error scales in the last three rows of Table 2. However, qualitatively the reconstructions are still accurate, as shown in Fig. 8.

This set of experiments shows that an AE-based model trained on SURREAL does not generalize well. In fact, the last 4 columns for the FAUST experiments show better reconstruction accuracy than the others, meaning that our learning model based on a parametric latent space (*S* and *SX*) is preferable in an out-of-distribution scenario.

On the other hand, the AE-based model is more appropriate whenever the input spectra are sampled from the same distribution as the training data, which is characteristic of encoder-decoder models. This is confirmed by the SURREAL tests in the Table, where $O_{30}$ outperforms all the SMPL-X based models by a large gap.

### Timing and Implementation Details

The experiments were run on a i9-9820X 3.30GHz CPU, with 32GB of RAM and a RTX 2080 Ti GPU. In general, the runtime depends on the number of vertices; for the data we used in our tests, on average we observed that an epoch requires 20 to 30 seconds. We used fewer vertices for the PointNet version of the network to compensate the computational cost of Chamfer distance computation. In our configuration, a full training requires 10 to 12 hours without any ad-hoc optimization (e.g., early stopping). Our code is publicly available at https://git.io/JGJWE.

## Application: Disentanglement

Our model naturally provides a tool to investigate the relationship between intrinsic and extrinsic geometric properties of the shapes being analyzed. In particular, given a latent vector $\mathbf {v}$ representing a shape, our model provides two differentiable maps taking $\mathbf {v}$ as input (Fig. 4):

- the decoder *D* between $\mathbf {v}$ and the extrinsic geometry of the shape, represented as vertex coordinates *V*;
- the network $\rho$, that maps $\mathbf {v}$ to the Laplacian spectrum, which is an intrinsic quantity widely used as a proxy for the shape metric.

These two maps allow us to locally separate between extrinsic and intrinsic shape information. Specifically, we can seek for shape deformations directly in the latent space, driven by either *D* or $\rho$. We first illustrate this mathematically, and then give concrete examples in the following.

Starting from any given latent vector $\mathbf {v}$, we can deform the corresponding shape $\mathcal {X}$ by moving $\mathbf {v}$ in the direction $\mathbf {d}$ that minimizes (or maximizes) the variation in the Laplacian spectrum. This is done by considering the Jacobian matrix of the network $\rho$, which we call $J_{\rho }$. The direction $\mathbf {d}$ of minimum (maximum) variation of $\mathrm {Spec}(\mathcal {X})$ is then given by the right-singular vector of $J_{\rho }$ corresponding to the smallest (largest) singular value, as explained in Section 7 of the Supplementary material. Thus, we can take an infinitesimal step along $\mathbf {d}$ by the update rule $\mathbf {v} \mapsto \mathbf {v} + \alpha \mathbf {d}$, with small $\alpha$.

**Fig. 10:**
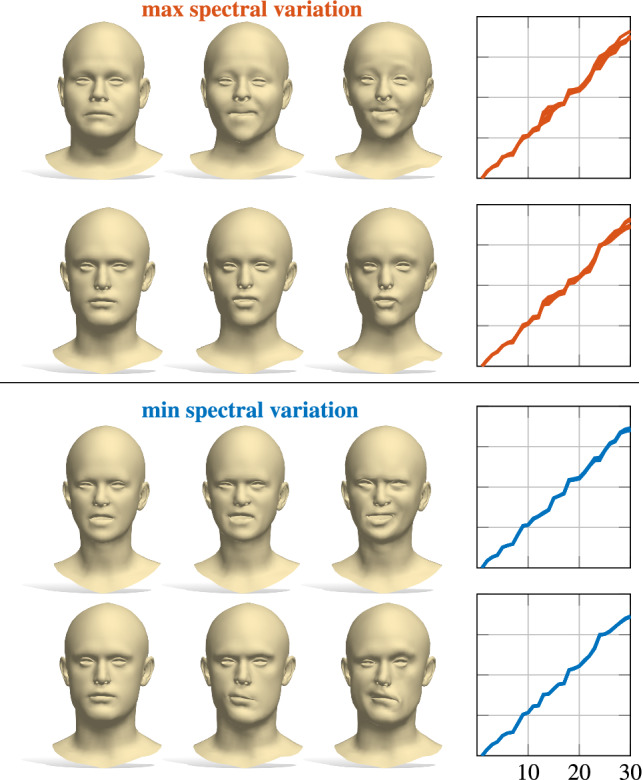
Latent space exploration using the spectral prior. Upper block of two rows: Examples of paths obtained maximizing the eigenvalues variation. The faces change both identities and poses. Lower block: Paths obtained moving towards the direction of minimum spectral variation. Shapes go through isometries (change of facial expression). On the right, the eigenvalues recomputed on each shape of the row are reported

In the case of deformable shapes as the ones of CoMA Ranjan et al. (2018), this results in the ability to continuously deform a shape while keeping its metric unchanged, *i*.*e*., to generate isometries. Examples of shapes generated according to this criterion are reported in Figure 10. As we can see, minimizing the spectral variation leads to approximately isometric deformations, resulting in a change of facial expression of the shapes, while maximizing the spectral variation induces a change in both their pose and identity.

**Fig. 11:**
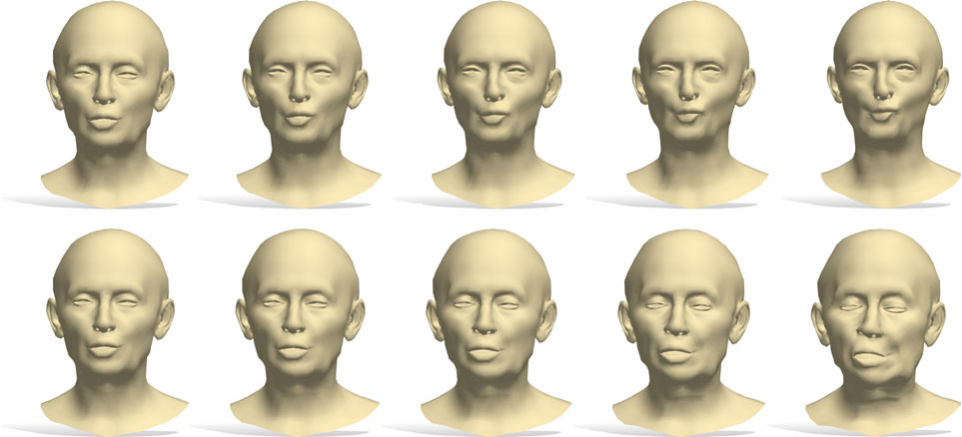
Latent space exploration using the spectral prior. First row is obtained maximizing intrinsic variation while keeping vertices as constant as possible, resulting in change of identity. Conversely, second row is maximum extrinsic variation and minimum variation of eigenvalues, inducing a change of pose

Alternatively, we can find the deformation of $\mathcal {X}$ that changes the intrinsic metric while preventing its extrinsic distortion from being too large. This means to update $\mathbf{v}$ by maximizing the spectral variation and, at the same time, keeping the decoded shape vertices *V* as constant as possible. Conversely, we could enhance the extrinsic distortion in isometric deformations, in order to obtain more pronounced changes of pose than the ones in Figure 10. Similarly to the previous case, both deformations can be achieved considering $J_{\rho }$ and the Jacobian of the decoder, see Supplementary for the details. Therefore, two additional types of latent space exploration paths driven by the spectral prior are possible: maximum spectral variation plus minimum extrinsic variation and vice-versa. Examples of these latent space explorations on CoMA are reported in Figure 11. They should correspond to the change of pose and change of identity respectively. We stress that such paths should emulate a change of pose/identity in an approximate way, but are not expected to produce high quality shape animations. In fact, we move in the latent space making small steps around the latent vector of an initial shape, but we are not guaranteed to be in the vicinity of a good solution in the first place. More visually pleasant solutions might be achieved via further post-processing in the vertex space.

## Application: Shape correspondence

An important application in the field of 3D shape analysis is establishing point-to-point correspondence between objects. In particular, given two shapes $\mathcal {X}$ and $\mathcal {Y}$, we aim to find a map $T_{\mathcal {X}\mathcal {Y}}: \mathcal {X} \xrightarrow {} \mathcal {Y}$ that associates for each point of the first shape a point of the latter. In this application, we exploit two of the main advantages of our method: the capability to recover a geometry from its spectrum, and the natural order of points provided by the decoder. Given two input shapes $\mathcal {X}$ and $\mathcal {Y}$ with their spectra $\lambda _{\mathcal {X}}$ and $\lambda _{\mathcal {Y}}$, we can approximate them computing $\mathcal {S}_{\mathcal {X}} = \mathbf {D}(\pi (\lambda _{\mathcal {X}}))$ and $\mathcal {S}_{\mathcal {Y}} = \mathbf {D}(\pi (\lambda _{\mathcal {Y}}))$. Being the outputs of our network discretized by a common template, we naturally obtain a correspondence between $\mathcal {S}_{\mathcal {X}}$ and $\mathcal {S}_{\mathcal {Y}}$. Given this correspondence, we can solve for the map $T_{\mathcal {X}\mathcal {Y}}$ in an alternative way: (1) we estimate $T_{\mathcal {X}\mathcal {S}_{\mathcal {X}}}$ and $T_{\mathcal {S}_{\mathcal {Y}}\mathcal {Y}}$, which are easier to compute; (2) we compose these two maps via $T_{\mathcal {S}_{\mathcal {X}}\mathcal {S}_{\mathcal {Y}}}$ that is given by construction; (3) the composition $T_{\mathcal {X}\mathcal {S}_{\mathcal {X}}} \circ T_{\mathcal {S}_{\mathcal {X}}\mathcal {S}_{\mathcal {Y}}} \circ T_{\mathcal {S}_{\mathcal {Y}}\mathcal {Y}}$ finally yields the desired correspondence. We consider two different settings for this problem. *Single-pose* matching, where we consider two objects that share the same pose reconstructed by our model; *Multi-pose* matching, where the two geometries have different poses from the one seen at training time. We show how our approach helps in both these settings.

**Table 3:** Quantitative evaluation for the non-rigid shape matching application, averaged over 10 shape pairs

FEM3	**Ours**	FMAP	**Ours**+ZO	FMAP+ZO
2-20	2.09e-2	2.46e-2	6.37e-3	6.58e-3
2-100	3.00e-2	3.57e-2	5.64e-3	9.41e-3
5-20	2.11e-2	2.41e-2	6.28e-3	6.71e-3
5-100	8.00e-3	9.30e-3	5.64e-3	9.41e-3

The results are the average geodesic errors reported in centimeters. Each row represents a specific experiment varying the number of landmarks (2 or 5), and the size of the estimated functional map (20 or 100)

**Fig. 12:**
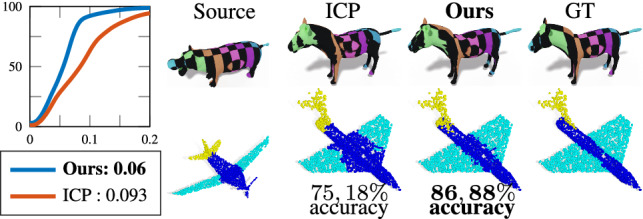
On the left, quantitative evaluation of matching (Kim et al. 2011) between 100 pairs of animals. On the right, a qualitative comparison on texture and segmentation transfer

**Single-pose** In this setting, $\mathcal {X}$, $\mathcal {Y}$ and $\mathcal {S}_{\mathcal {X}}$, $\mathcal {S}_{\mathcal {Y}}$ are all in the same pose and location in 3D space, thus we can establish a mapping between each input and its reconstruction via nearest-neighbor assignment in 3D. Then, exploiting the common discretization of $\mathcal {S}_{\mathcal {X}}$ and $\mathcal {S}_{\mathcal {Y}}$, we obtain a sparse correspondence between the two original shapes. In the case of meshes, we then extend the sparse matching on all the surface using the functional maps framework (Ovsjanikov et al. 2012), while for point clouds we just propagate it by nearest-neighbor. We remark that we obtain the correspondence automatically from the spectra of the shapes. We perform a quantitative evaluation on SMAL (Zuffi et al. 2017), testing on 100 non-isometric pairs of animals from different classes. As a baseline we consider ICP (Besl and McKay 1992) to rigidly align the two shapes (100 iterations), followed by nearest-neighbor assignment to obtain a correspondence. Two applications that benefit from our approach are texture and segmentation transfer; we tested them respectively on animals and segmented ShapeNet  (Yi et al. 2017). See Fig. 12 and the supplementary for further details.

**Fig. 13:**
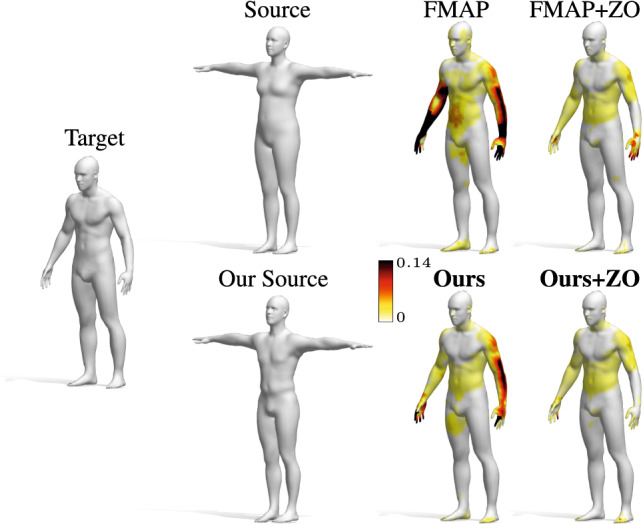
Qualitative comparison of non-rigid shape matching. Top row: The source shape is a different subject than the target, and is in a different pose; the last two columns show the matching results obtained with standard methods. Bottom row: The source shape is recovered from the spectrum of the target shape by using our model $O_{30}$, making the correspondence problem easier to solve. The geodesic error (in cm) is encoded by color, growing from white to dark red

**Multi-pose matching** We now consider two shapes that do not share the same spatial pose, have a different connectivity, and are also affected by non-rigid deformations. To find a correspondence, we use again the functional maps framework (Ovsjanikov et al. 2012) (FMAP). Such framework entirely relies on the intrinsic geometry of the shapes, and so it is robust to nearly-isometric changes of the subject, however, it suffers in the presence of non-isometric deformations. Here we consider 10 shape pairs $(\mathcal {X}$, $\mathcal {Y})$, where $\mathcal {X}$ is one of the 10 different human identities from FAUST, and $\mathcal {Y}$ is the SMPL template. Each shape $\mathcal {X}$ is non-isometric and in a different pose than $\mathcal {Y}$. With our model, we compute $\mathcal {S}_{\mathcal {X}}$ as a mesh with the same connectivity and pose of SMPL that is isometric to $\mathcal {X}$, while we let $\mathcal {S}_{\mathcal {Y}} = \mathcal {Y}$ be the SMPL template. Then, we compute the correspondence between $\mathcal {X}$ and $\mathcal {S}_{\mathcal {X}}$ via the FMAP implementation of Nogneng and Ovsjanikov (2017), and obtain a matching between $\mathcal {X}$ and $\mathcal {Y}$ by composition as explained above. We perform this test while varying two important parameters of FMAP: the number of ground-truth landmarks used as probe functions (2 or 5), and the dimension of the functional correspondence matrix (20 or 100). To highlight the benefits introduced by our approach, we compare against the baseline obtained applying the framework (Nogneng and Ovsjanikov 2017) directly to the shape pair $(\mathcal {X}$, $\mathcal {Y})$. In the second and third columns of Table 3 we report the results of our method and the baseline respectively. We notice that by producing a more isometric template, we obtain a significant improvement in performance. Furthermore, in the last two columns, we report the results obtained with the ZoomOut refinement algorithm (Melzi et al. 2019), applied with the parameters proposed in the original paper. This procedure promotes isometric maps, which makes our contribution even more crucial. A qualitative comparison is depicted in Fig. 13.

## Additional applications

**Fig. 14:**
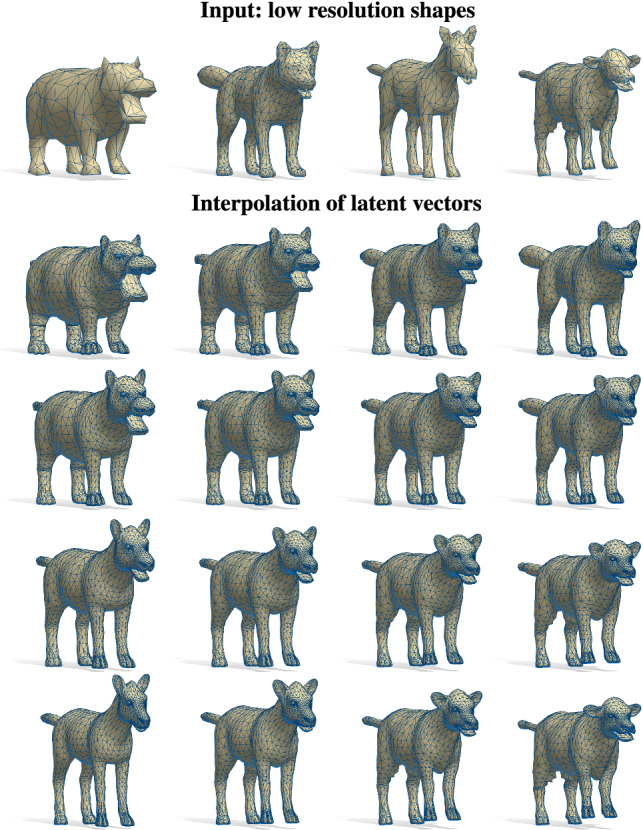
Latent space interpolation of four low-resolution shapes with different mesh connectivity (top row, unseen at training time). The spectra of the input shapes are mapped via $\pi$ to the latent space, where they are bilinearly interpolated and then decoded to $\mathbb {R}^3$. The reconstructions of the four input shapes are depicted at the four corners of the $4\times 4$ grid

**Fig. 15:**
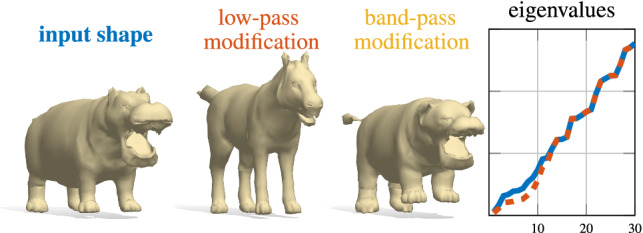
Exploring the space of shapes in real time via manipulation of the spectrum. The low-pass modification (middle) decreases the first 12 eigenvalues of the input shape; the band-pass modification (right) amplifies the last 12 eigenvalues. The damping of low eigenvalues leads to more pronounced geometric features (e.g. longer legs and snout), while amplification of mid-range eigenvalues affects the high-frequency details (e.g. the ears and fingers); see the supplementary video for a wall-clock demo

**Fig. 16:**
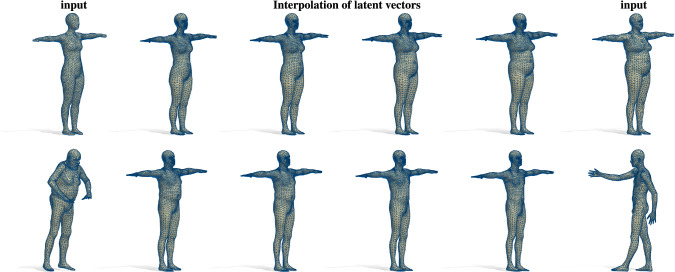
Latent space interpolation for the human bodies category. On the left and right of each row, we depict the input shapes with different connectivity. We map their spectra in the latent space, where we linearly interpolate and then decode them via $\mathbf {D}$, thus generating the shapes in the five intermediate columns. First row: The input shapes have been remeshed and have never been seen during training (SURREAL dataset). Second row: The shapes have been remeshed and have different poses, and belong to subjects that are not in the space spanned by the SMPL morphable model (FAUST dataset). This shows the robustness of our method to different subjects and different poses

Our general model enables several additional applications, by exploiting the connection between spectral properties and shape generation. Due to the limited space, we collect in the supplementary materials the details of the training and test sets and the parameters used in our experiments.

### Shape exploration

The results of Sects. 5 and 6 suggest that eigenvalues can be used to drive the exploration of the AE’s latent space toward a desired direction. Another possibility is to regard *the eigenvalues themselves* as a parametric model for isometry classes, and explore the “space of spectra” as is typically done with latent spaces. Our bi-directional coupling between spectra and latent codes makes this exploration feasible, as remarked by the following property:

#### Property 1

Latent space connections provide both a means for **controlling** the latent space, and vice-versa, enable **exploration** of the space of Laplacian spectra.

**Fig. 17:**
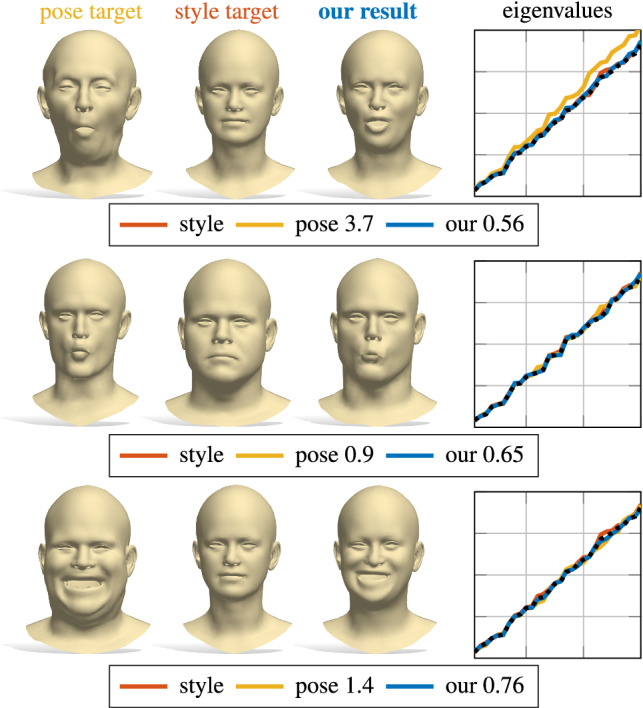
Examples of style transfer. The target style (middle) is applied to the target pose (left) by solving problem (9) and then decoding the resulting latent vector, obtaining the result shown on the right. For each example we also report the corresponding eigenvalue alignment (rightmost plots). The black dotted line is the image of $\rho$. The numbers in the legend denote the distance from the target “style” spectrum to the source pose and to our generated shape; a small number suggests that the generated shape is a near-isometry of the style target

**Fig. 18:**
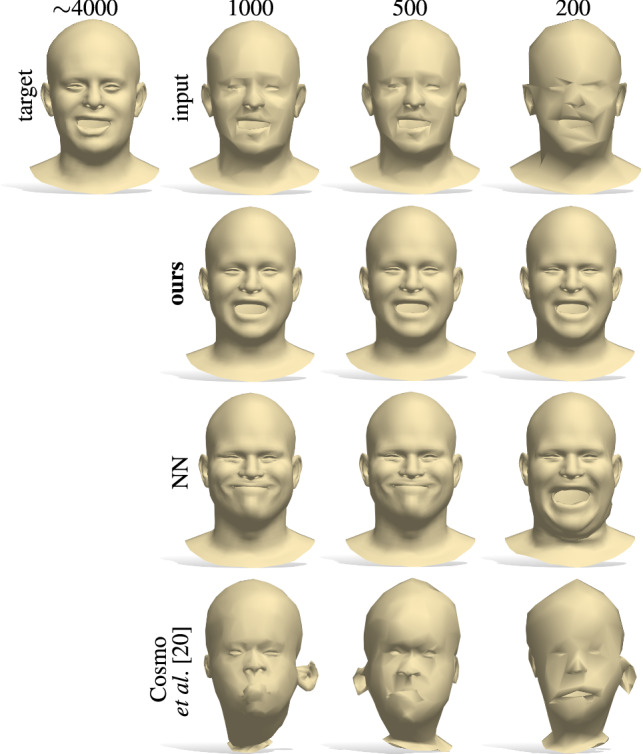
Mesh super-resolution for inputs at decreasing resolution (top row, left to right). Our method fits closely the original input shapes (top left), while other approaches either predict the wrong pose (NN baseline) or generate an unrealistic shape (Cosmo et al. (2019))

Since eigenvalues change continuously with the manifold metric (Bando and Urakawa 1983), a small variation in the spectrum will give rise to a small change in the geometry. We can visualize such variations in shape directly, by first deforming a given spectrum (*e*.*g*., by a simple linear interpolation between two spectra) to obtain the new eigenvalue sequence ${\varvec{\mu }}$, and then directly computing $D(\pi ({\varvec{\mu }}))$.

In Fig. 14 we show a related experiment. Here we train the network on 4,430 animal meshes generated with the SMAL parametric model following the official protocol (Zuffi et al. 2017). Given four *low-resolution* shapes $\mathcal {X}_i$ as input, we first compute their spectra $\mathrm {Spec}(\mathcal {X}_i)$, map these to the latent space via $\pi (\mathrm {Spec}(\mathcal {X}_i))$, perform a bilinear interpolation of the resulting latent vectors, and finally reconstruct the corresponding shapes. We perform the same experiment on the human bodies category by exploiting the model $O_{30}$. In Fig. 16, we consider two meshes from the SURREAL test set and two shapes from FAUST dataset. All the input shapes have been remeshed with different densities. The linear interpolation of the latent vectors obtained through $\pi$ produces meaningful intermediate steps encoding the main intrinsic variation of the subjects involved. We remark that the pose variations of a human shape are close to isometric deformations and therefore do not affect the Laplacian spectrum. For this reason, it is not possible to retrieve the pose of a human body from its spectrum. In this spirit, we trained our model only on shapes in T-Pose, motivating the pose of the interpolation steps in Fig. 16. Furthermore, our method is robust to changes in connectivity, extrinsic pose and embedding (note the rigid rotation between the initial and final input shapes in the second row).

Finally, in Fig. 15 we show an example of interactive spectrum-driven shape exploration for the animals class. Given a shape and its Laplacian eigenvalues as input, we navigate the space of shapes by directly modifying different frequency bands with the aid of a simple user interface. The modified spectra are then decoded by our network in *real time*. The interactive nature of this application is enabled by the efficiency of our shape from spectrum recovery (obtained in a single forward pass) and would not be possible with previous methods (Cosmo et al. 2019) that rely on costly test-time optimization. We refer to the accompanying video and the supplementary materials for additional illustrations.

**Fig. 19:**
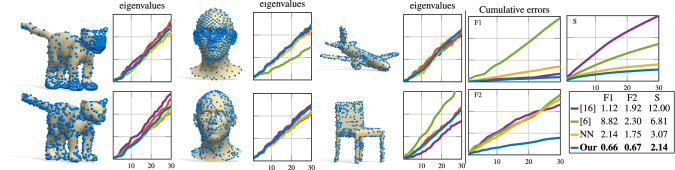
Qualitative and quantitative evaluation of point cloud spectra estimation. On the left we show the qualitative comparison for different samplings on three classes (animals, human faces and objects). We show the eigenvalues estimations alongside the input point cloud (depicted as surface samplings), and the ground truth spectrum (in red). On the last two columns, we report the average cumulative error curves evaluated on the FLAME dataset for the two different distributions (F1 and F2) and on ShapeNet (S)

### Style transfer

As shown in Fig. 1, we can use our trained network to transfer the style of a shape $\mathcal {X}_\mathrm {style}$ to another shape $\mathcal {X}_\mathrm {pose}$ having both a different style and pose. This is done by a search in the latent space, phrased as:

9 $$\begin{aligned} \min _{\mathbf {v}} \Vert \mathrm {Spec}(\mathcal {X}_\mathrm {style}) - \rho (\mathbf {v}) \Vert _2^2 + w \Vert \mathbf {v} - E(\mathcal {X}_\mathrm {pose}) \Vert _2^2 \end{aligned}$$

Here, the first term seeks a latent vector whose associated spectrum aligns with the eigenvalues of $\mathcal {X}_\mathrm {style}$; in other words, we regard style as an intrinsic property of the shape, and exploit the fact that the Laplacian spectrum is invariant to pose deformations. The second term keeps the latent vector close to that of the input pose (we initialize with $\mathbf {v}_\mathrm {init}=E(\mathcal {X}_\mathrm {pose})$). We solve the optimization problem by back-propagating the gradient of the cost function of Eq. (9) with respect to $\mathbf {v}$ through $\rho$.

The sought shape is then given by a forward pass on the resulting minimizer. In Fig. 17, we show four examples (others can be found in the supplementary material). We emphasize here that the style is purely encoded in the input eigenvalues, therefore it does not rely on the test shapes being in point-to-point correspondence with the training set. This leads to the following:

#### Property 2

Our method can be used in a **correspondence-free** scenario. By taking eigenvalues as input, it enables applications that traditionally require a correspondence, but side-steps this requirement.

This observation was also mentioned in other spectrum-based approaches (Cosmo et al. 2019; Rampini et al. 2019). However, the data-driven nature of our method makes it more robust, efficient and accurate, therefore greatly improving its practical utility.

### Super-resolution

A key feature that emerges from the experiment in Fig. 14 is the perfect reconstruction of the low-resolution shapes once their eigenvalues are mapped to the latent space via $\pi$. This brings us to a fundamental property of our approach:

#### Property 3

Since eigenvalues are largely **insensitive to mesh resolution and sampling**, so is our trained network.

This fact is especially evident when using cubic FEM discretization, as we do in all our tests, since it more closely approximates the continuous setting and is thus much less affected by the surface discretization.

***Remark.*** It is worth mentioning that existing methods can employ cubic FEM as well; however, this soon becomes prohibitively expensive due to the differentiation of spectral decomposition required by their optimizations (Cosmo et al. 2019; Rampini et al. 2019).

These properties allow us to use our network for the task of mesh super-resolution. Given a low-resolution mesh as input, our aim is to recover a higher resolution counterpart of it. Furthermore, while the input mesh has *arbitrary* resolution and is unknown to the network (and a correspondence with the training models is *not* given), an additional desideratum is for the new shape to be in dense point-to-point correspondence with models from the training set. We do so in a single shot, by predicting the decoded shape as:

10 $$\begin{aligned} \mathcal {X}_\mathrm {hires} = D(\pi (\mathrm {Spec}(\mathcal {X}_\mathrm {lowres})))\,. \end{aligned}$$

This simple approach exploits the resolution-independent geometric information encoded in the spectrum along with the power of a data-driven generative model.

In Fig. 18 we show a comparison with nearest-neighbors between eigenvalues (among shapes in the training set), and the isospectralization method of Cosmo et al. (2019). Since we can exploit the cubic FEM, which is less sensitive to the different resolutions, our solution closely reproduces the high-resolution target. Isospectralization correctly aligns the eigenvalues, but it recovers unrealistic shapes due to ineffective regularization. This phenomenon highlights the following

#### Property 4

Our data-driven approach replaces ad-hoc regularizers, that are difficult to model axiomatically, with **realistic priors** learned from examples.

This is especially important for deformable objects; shapes falling into the same isometry class are often hard to disambiguate without using geometric priors.

### Estimating point cloud spectra

As an additional experiment, we show how our network can directly predict Laplacian eigenvalues for unorganized point clouds. This task is particularly challenging due to the lack of a structure in the point set, and existing approaches such as (Clarenz et al. 2004; Belkin et al. 2009) often fail at approximating the eigenvalues of the underlying surface accurately. The difficulty is even more pronounced when the point sets are irregularly sampled, as we empirically show here. In our case, estimation of the spectrum boils down to the single forward pass:

11 $$\begin{aligned} \widetilde{\mathrm {Spec}}(\mathcal {X})=\rho (E(\mathcal {X}))\,. \end{aligned}$$

To address this task we train our network by feeding unorganized point clouds as input, together with the spectra computed from the corresponding meshes (which are available at training time). As described in the supplementary materials, for this setting we use a PointNet (Qi et al. 2017) encoder and a fully connected decoder, and we replace the reconstruction loss of Eq. (5) with the Chamfer distance. This application highlights the generality of our model, which can accommodate different representations of geometric data.

We consider two types of point clouds: (1) with similar point density and regularity as in the training set (shown in the supplementary materials), and (2) with randomized non-uniform sampling. We compare the spectrum estimated via $\rho (E(\mathcal {X}))$ to axiomatic methods (Clarenz et al. 2004; Belkin et al. 2009), and to the NN baseline (applied in the latent space); see Fig. 19. The qualitative results are obtained by training on SMAL (Zuffi et al. 2017) (left), COMA (Ranjan et al. 2018) (middle) and ShapeNet watertight  (Huang et al. 2018) (right). To highlight its generalization capability, the network trained on COMA is tested on point clouds from the FLAME dataset, while on ShapeNet we consider 4 different classes (airplanes, boats, screens and chairs). We compute the cumulative error curves of the distance between the eigenvalues from the meshes corresponding to the test point clouds. The mean error across all test sets is also reported in the legend. Our method leads to a significant improvement over the closest state-of-the-art baseline (Belkin et al. 2009).

## Conclusions

We introduced the first data-driven method for shape generation from Laplacian spectra. Our approach consists in enriching a standard AE with a pair of cycle-consistent maps, associating ordered sequences of eigenvalues to latent codes and vice-versa. This explicit coupling brings forth key advantages of spectral methods to generative models, enabling novel applications and a significant improvement over existing approaches. These maps provide an effective tool for a geometrically meaningful exploration of the latent space, and further allow to disentangle the intrinsic from the extrinsic information of the shapes. Our main limitations are shared with other spectral methods in the computation of a robust Laplacian discretization. Adopting the recent approach (Sharp et al. 2019) for such borderline cases is a promising possibility. Further, while the Laplacian is a classical choice due to its Fourier-like properties, the spectra of other operators with different properties may lead to other promising applications. Finally, considering more complex and structured generative models (e.g. probabilistic or hierarchical ones (Gao et al. 2019)) in our pipeline may give rise to promising directions for further investigation.

## Supplementary Information

Below is the link to the electronic supplementary material.

Supplementary material 1 (pdf 29198 KB)

Supplementary material 2 (mp4 1170 KB)

## Footnotes

1 Similarly to Cosmo et al. (2019) we use homogeneous Neumann boundary conditions; see Eq. (2), where $\hat{n}(x)$ denotes the outward normal to the boundary.
