# Supplementary material for: Spectral Shape Recovery and Analysis Via Data-driven Connections
Source: Int J Comput Vis. 2021 Jul 22;129(10):2745–60. doi: 10.1007/s11263-021-01492-6 (PMC8550494; doi:10.1007/s11263-021-01492-6)
Supplement: Supplementary file 1 — Supplementary material 1 (pdf 29198 KB) [file 11263_2021_1492_MOESM1_ESM.pdf]

Noname manuscript No.
(will be inserted by the editor)

Spectral shape recovery and analysis via data-driven connections – Supplementary Materials

Riccardo Marin · Arianna Rampini · Umberto Castellani · Emanuele Rodolà · Maks Ovsjanikov · Simone Melzi

Received: date / Accepted: date

Abstract In this document we collect some additional details about the proposed method, architecture and results, that due to lack of space were not included in the main manuscript.

1 Architecture

Meshes. When $\mathcal{X}$ is discretized as a mesh (typical in graphics and geometry processing applications), the AE architecture is a multilayer perceptron (MLP) with a bottleneck and dimensions: $n \times 3 \to 300 \to 200 \to 30 \to 200 \to n \times 3$; each layer, except for the last one, is followed by tanh activation. The maps π and ρ are both parametrized by a MLP with dimensions: $k \to 80 \to 160 \to 320 \to 640 \to 320 \to 160 \to 80 \to k$; each layer, except for the last one, is followed by batch norm and SeLu activation. We use batch size 16, and the Adam optimizer with learning rate equal to 10^{-4}. Note that we also use this architecture for 2D contours, which are discretized as (2-regular) cycle graphs.

Unorganized point clouds. In this case we do *not* assume the training data to have a consistent vertex labeling. To tackle this setting, we use a PointNet [7] encoder and a fully connected decoder[1]; we use the MLP from the mesh case for the decoder. The encoder consists in a shared MLP network on each point with layer output sizes 64 and 128; each layer is followed by batch norm. A maxpool layer is then used to output a 128-dimensional vector, which is reduced with a tanh-activated MLP to dimensions: $128 \to 64 \to 30$.

The full implementation of the proposed architecture and the data that we used in our experiments can be retrived here: [1] .

2 Details on the latent space connections for parametric models

A popular way to produce datasets (and also the primary approach we used in our work) is synthetizing them directly from parametric models. These models generate different instances of the same class by modifying some attributes of a common template. In general, a subset β of their parameters are aimed to modify the characteristics of subject *identity.* One may wonder if it would be possible establishing the connection directly between the space of parameters of such models and the spectrum of the generated identities (as shown in Figure 5 in the main paper), without training an ad-hoc autoencoder (as shown in Figure 4 in the main paper). To analyze this aspect, we considered two morphable models for human bodies. The first is the gender-neutral version of SMPL; the publicly available version of this model has ten identity parameters that modify the template $\mathbf{T}$ as a linear combination of the basis $\mathbf{S}$, producing a final vertex coordinates X.

$$\mathcal{X}_\beta = \mathbf{T} + S\beta. \tag{1}$$

We generated a dataset of pairs $(\beta, Spec(\mathcal{X}_\beta))$, and we look for a map between the parameters representation

Riccardo Marin · Arianna Rampini · Emanuele Rodolà · Simone Melzi
Sapienza University of Rome, Italy
E-mail: marin@di.uniroma1.it

Umberto Castellani
University of Verona, Italy

Maks Ovsjanikov
LIX, Ecole Polytechnique, IP Paris, France

[1] As suggested by the authors of [7] in `https://github.com/charlesq34/pointnet-autoencoder`

and the spectrum one. Since we have only 10 parameters, to maintain the bijective of the map we also consider the first 10 eigenvalues of the spectrum, obtaining a map between ten dimensional spaces (in the Table 2 on the main paper S_{10}). We also extended our analysis to other bandwidth. To do so, we rely on SMPL-X model, an advanced version of SMPL that generate shapes in an analogous way, but it can use up to 400 linear basis to generate identities; we use this model to generate maps between spaces at $15, 30$ and 60 dimensions (in the Table 2 on the main paper $SX_{15}, SX_{30}, SX_{60}$). SMPL-X has several other major differences from SMPL: it has two disconnected eyes, it comes from a composition of different models for head and hands (and so SMPL-X does not share the same triangulation of SMPL, i.e., they are not in correspondence), their templates are not aligned in the 3D space and also the parameters between SMPL and SMPL-X (both for identities and poses) are not interchangeable even for the parts in common. For this reason, in our evaluation, we removed the two eyes components, and we rely on a correspondence between SMPL-X and SURREAL given by a registration of T-Pose templates, following the procedure released by SMPL-X authors. While we think this lets us have models reliably aligned with ones from the SURREAL dataset, we think this parsing introduces some noises.

However, we would like to highlight that this is not a real limit, since in any case the direct map between parametric model parameters and the spectrum can be always computed. In fact:

$$\mathcal{X} = \mathbf{T} + \beta\mathbf{S} \tag{2}$$

$$\beta = \mathbf{S}^{-1}(\mathcal{X} - \mathbf{T}) \tag{3}$$

and with our model we have:

$$\mathbf{v} = E(\mathcal{X}), Spec(\mathcal{X}) = E(X), D \approx E^{-1} \tag{4}$$

the two maps between the morphable model parameters space and the spectrum can be easily recovered via composition. Hence, the map $\pi_S : Spec(\mathcal{X}) \to \beta$ is given by:

$$\beta = \mathbf{S}^{-1}(D(\pi(Spec(\mathcal{X}) - \mathbf{T}))) \tag{5}$$

and the map $\rho_S : \beta \to Spec(\mathcal{X})$ can be recovered by:

$$Spec(\mathcal{X}) = \rho(E(\mathbf{S}\beta + \mathbf{T})). \tag{6}$$

3 Additional analysis of increasing bandwidth

We performed an experimental analysis for different values of k (the number of Laplacian eigenvalues given as input). This parameter affects both the amount of information used by the network (*i.e.*, more eigenvalues) and the capability of the learned representation (*i.e.*, higher-dimensional latent space). We evaluate our model with $k = 15, k = 30$ and $k = 60$ on the shape-from-spectrum reconstruction task, using exactly the same dataset and experimental setup as in Table 1 of the main manuscript.

The quantitative results are reported in Table 1. While cutting k by half produces a sensible performance degradation, we observed that doubling it achieves just slightly better results. For this reason, in our experiments we settled for a value of $k = 30$. From a qualitative perspective, our results suggest that without changing other architecture details, a larger value for k can achieve better precision on the high-frequency details (*e.g.*, the pose of the mouth); as shown in Fig. 1.

	full res	1000	500	200
$k = 30$	1.61	1.62	**1.71**	2.13
$k = 15$	3.74	3.78	3.72	3.59
$k = 60$	**1.60**	**1.52**	1.79	**2.05**

Table 1 Shape-from-spectrum reconstruction comparisons with different k trained for the same number of epochs; we report average error over 100 shapes of an unseen subject from the COMA dataset [8]. All errors must be rescaled by 10^{-5}.

4 Laplace-Beltrami operator discretization

One of the advantages of our pipeline is that we can use the *cubic* FEM (*e.g.*[9, Sec. 4.1]) to discretize the Laplace-Beltrami operator, virtually without any additional cost if compared with the linear finite elements. The cubic FEM yields a more accurate discretization and improves the quality of the results produced by our pipeline. In Fig. 2 and in Table 2 in the main paper, we collect additional comparisons between the two different discretizations.

5 Training and test details

Here we summarize the data involved in the trainings and in the experiments. In the code that we will release upon acceptance, we will attach pre-processing scripts and when possible the data used to permit replication.

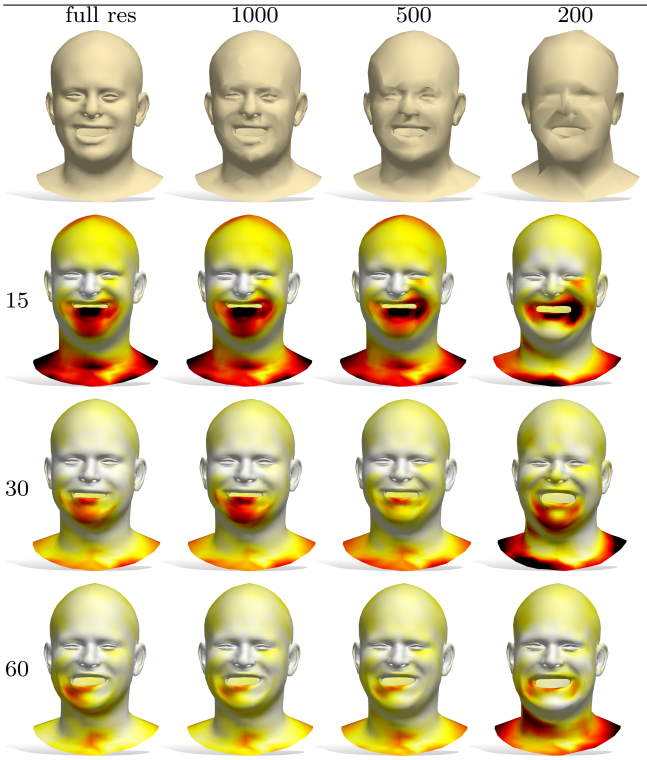

Fig. 1 Examples of shape-from-spectrum reconstructions at different values for the spectral bandwidth k; see Table 1 for a quantitative evaluation.

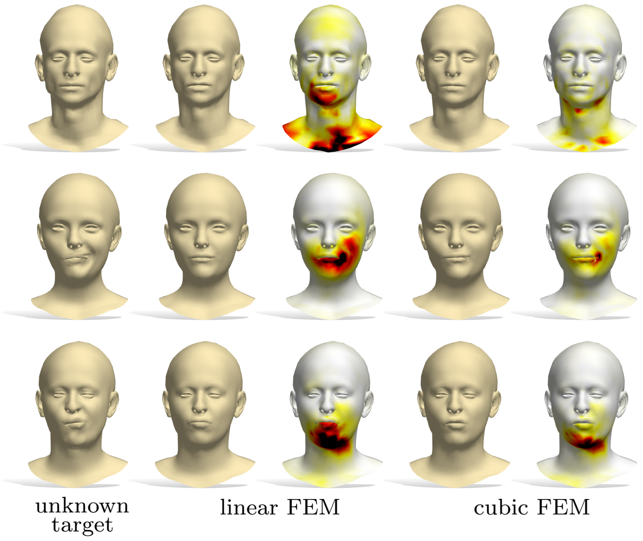

Fig. 2 Additional comparisons (one per row) between the use of eigenvalues of the Laplacian discretized with linear FEM or with cubic FEM in our shape-from-spectrum pipeline. The heatmap encodes point-wise reconstruction error, growing from white to dark red. "Unknown target" in the left-most column refers to the source shape from which the eigenvalues are computed.

Overall, we perform five different trainings: for CoMa and SMAL datasets we train the dense and the PointNet version of our Network on the same set of data. We

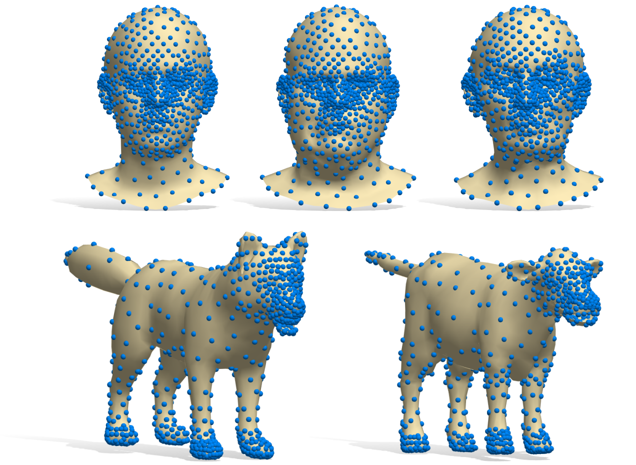

Fig. 3 Examples of point clouds used to train the PointNet version of our network in case of CoMa (first row) and SMAL (second row).

obtained the point clouds for PointNet trainings with a sparse sampling over the original meshes (the number of sampled point is around 20% of the mesh vertices at full resolution): some examples of training point clouds are shown in Figure 3. For the ShapeNet dataset, only the PointNet version is trained using 500 points for shape. The applications use these five models, and we do not do any ad-hoc optimization for different tasks.

Concerning the involved training sets:

CoMa. We use 1,853 shapes randomly picked between 11 subjects from the CoMa dataset [8] (168 shapes for each subject), leaving out the 12th subject for testing purpose.

SMAL. We use 4,430 animal meshes generated with the SMAL [10] generative model, among all the five possible classes. Thanks to the method explained in the original paper, we can cluster them in the correct races while we explore the inter-class variety.

ShapeNet. We would remark that ShapeNetCore dataset [3] is not practical for standard spectral analysis since the models consist of non-manifold disconnected pieces. For this reason, we used a processed watertight version of it [5]. From it, we pruned the meshes that still have artifacts (e.g. disconnected components). Finally, we remeshed the remaining ones to have at maximum 10K vertices. For training, we relies on four different classes: airplanes, boats, screens, and chairs. We pick 2,047 meshes from these classes, among the ones with a more reliable spectrum (i.e. removing outliers case). This last property was not considered for the test set, to challenge ourselves also against non-standard models (as you can see in Figure 11 of the main manuscript, where the target airplane has a tail's part missing).

Concerning the quantitative experiments:

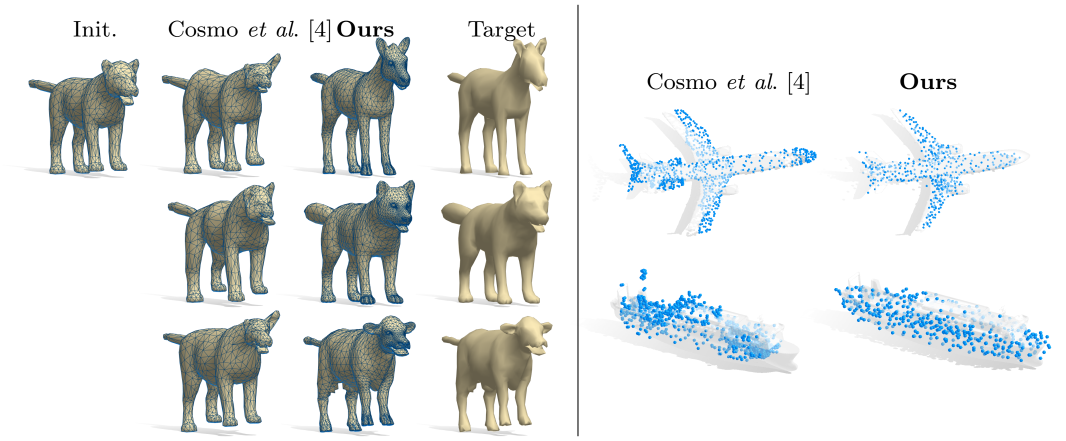

Fig. 4 Comparison in shape from spectrum estimation between isospectralization [4] and our method in the case of meshes (left) and point clouds (right). The *Init.* shape on the left is the neutral shape of the SMAL[10] generative model used as initialization of isospectralization algorithm for animals. The target shapes in the point cloud case are shown under the point clouds.

Shape from spectrum. This experiment was performed on a total of 400 shapes; 100 shapes of the unseen subject from the CoMa dataset at four different resolutions.

Estimating point cloud spectra. Here we consider a total of 1,186 models for the test: 393 shapes of different subjects from FLAME [6] dataset at two different samplings, and 400 shapes from ShapeNet from the four classes.

Shape matching. Finally, for the shape matching, we consider 100 couples of random animals of different species. These animals are generated with the same method of the above training set, but with higher variance in the generative space of SMAL. In this way, the testset presents more extreme characteristics of the ones seen at training time. For matching, we mainly consider extreme cases where high non-isometric deformation occurs (e.g.as shown in Figure 11 between hippos and horses). As a final comment, we would emphasize that our results against Nearest-Neighbor suggest our generalization capability and the real challenge of our testsets.

6 Shape from spectrum

In the task of recovering shapes from Laplacian spectra we compared with the *isospectralization* approach introduced in [4]. In Fig. 4, we report some qualitative examples from SMAL dataset [10] and ShapeNet dataset [5]. For SMAL, both the unknown shape to be recovered and our reconstruction are meshes (left panel of Fig. 4); for objects from ShapeNet, our network has been trained on unorganized sparse point clouds, so the reconstruction in this case is a point cloud as well (right panel of Fig. 4). Our method recovers shape from spectrum in a *single forward pass*, while isospectralization *deforms* an initial shape such that its spectrum align the one of a target shape using geometric regularizers. As initialization for isospectralization we used the template of SMAL [10] for animals, and the closest (in terms of distance between the eigenvalues) shape from our training set for objects of ShapeNet. Even so, isospectralization produces unrealistic instances or fails to capture the geometry of the target compared to our method.

7 Latent space exploration tools

In the main manuscript, we use the differentiable map between latent vectors and eigenvalues to perform a geometric-based exploration of the latent space. This is done by minimizing or maximizing the spectral variation while moving in the latent space as explained in the following.

Given a differentiable function $\mathbf{f} : \mathbb{R}^d \to \mathbb{R}^D$ and a point $\mathbf{x_0} \in \mathbb{R}^d$, the direction in $\mathbb{R}^d$ towards which the variation of the L_2 norm $\|\mathbf{f}\|$ is maximum is given by the principal right-singular vector of the Jacobian of $\mathbf{f}$ in $\mathbf{x_0}$. Indeed, considering the first order Taylor expansion:

$$\mathbf{f}(\mathbf{x_0} + \mathbf{dx}) \simeq \mathbf{f}(\mathbf{x_0}) + \mathbf{J}_0\mathbf{dx}$$

where $\mathbf{J_0}(i,j) = \partial f_i / \partial x_j$, we are looking for the vector $\mathbf{dx}$ which satisfies:

$$\max_{\mathbf{dx}\text{ s.t. }\|\mathbf{dx}\|=\epsilon} \|\mathbf{J}_0\mathbf{dx}\| \quad (7)$$

Using the singular value decomposition $\mathbf{J_0} = \mathbf{U\Sigma V}^T$, we can write $\mathbf{dx} = \mathbf{Vc}$ and (7) as:

$$\max_{\mathbf{c}\text{ s.t. }\|\mathbf{c}\|=\epsilon} \|\mathbf{U\Sigma c}\| = \max_{\mathbf{c}\text{ s.t. }\|\mathbf{c}\|=\epsilon} \|\sum_{i=1}^{d} \mathbf{u_i}\sigma_i c_i\|$$
$$= \max_{\mathbf{c}\text{ s.t. }\|\mathbf{c}\|=\epsilon} \sum_{i=1}^{d} \sigma_i^2 c_i^2$$

where $\mathbf{u_i}$ are the orthonormal left-singular vectors and $\sigma_i = \Sigma_{ii}$ are the singular values of $\mathbf{J_0}$. If σ_1 is the greatest singular value, the sought direction is $\mathbf{dx} = \epsilon\mathbf{v_1}$.

Similarly, the direction of minimum variation of $\|\mathbf{f}\|$ is given by the right-singular vector of $\mathbf{J_0}$ corresponding to the smallest singular value.

As discussed in the main manuscript, we are interested also in maximizing the spectral variation while keeping at the same time the extrinsic deformation as small as possible (or vice-versa) in order to disentangle the intrinsic and extrinsic properties of shapes. The relation between latent vectors and extrinsic geometry is given by another differentiable map, i.e. the decoder. In other words, we are given two differentiable functions $\mathbf{f}: \mathbb{R}^d \to \mathbb{R}^{D_1}$ and $\mathbf{g}: \mathbb{R}^d \to \mathbb{R}^{D_2}$ and we are interested in varying $\mathbf{f}$ while keeping $\mathbf{g}$ as constant as possible. Hence, we can maximize the quantity:

$$E(\mathbf{dx}) = \|\mathbf{f}(\mathbf{x_0}+\mathbf{dx}) - \mathbf{f}(\mathbf{x_0})\| - \|\mathbf{g}(\mathbf{x_0}+\mathbf{dx}) - \mathbf{g}(\mathbf{x_0})\|$$
$$= \|\mathbf{J}_0^{(f)}\mathbf{dx}\| - \|\mathbf{J}_0^{(g)}\mathbf{dx}\|$$

Using singular value decomposition as before

$$\mathbf{J_0^{(g)}} = \mathbf{U^{(g)}\Sigma^{(g)}V^{(g)}}^T$$
$$\mathbf{J_0^{(f)}} = \mathbf{U^{(f)}\Sigma^{(f)}V^{(f)}}^T$$
$$\mathbf{dx} = \mathbf{V^{(f)}c} = \mathbf{V^{(g)}c_g}$$

we obtain:

$$E(\mathbf{c}) =$$
$$\sum_{i=1}^{d}\Big((\sigma_i^{(f)})^2 c_i^2 - (\sigma_i^{(g)})^2 \sum_{j,k} (\mathbf{v}_i^{(f)}\cdot\mathbf{v}_j^{(g)})(\mathbf{v}_i^{(f)}\cdot\mathbf{v}_k^{(g)}) c_j c_k\Big)$$

Defining the $d \times d$ matrix

$$M_{j,k} = \delta_{j,k}\sigma_j^{(f)}\sigma_k^{(f)} - \sum_i \Big(\big(\sigma_i^{(g)}\big)^2 (\mathbf{v}_i^{(f)}\cdot\mathbf{v}_j^{(g)})(\mathbf{v}_i^{(f)}\cdot\mathbf{v}_k^{(g)})\Big)$$

we can rephrase the optimization problem as follows:

$$\max_{\mathbf{c}\text{ s.t. }\|\mathbf{c}\|=\epsilon} \mathbf{c^T M c}.$$

Thus the desired direction $\mathbf{dx}$ is given by the principal eigenvector of $\mathbf{M}$.

8 Super-resolution

Our trained network model is largely insensitive to mesh resolution and sampling as we show in our experiments in the main manuscript. This property makes our network appropriate for the task of mesh super-resolution. Given a low-resolution mesh as input, we can recover a higher resolution counterpart of the mesh that is also in dense point-to-point correspondence with models from the training set. We can do that in a single shot starting only from the spectrum of the input low-resolution mesh. In Fig. 5, we show a comparison with nearest-neighbors (NN) between eigenvalues (with shapes within the training set), and the *isospectralization* method of Cosmo *et al.* [4] for two different shapes from a subject that is not involved in the training of our network model.

In Fig. 6, we perform the same experiment on three different shapes never seen during the training, and belonging to a subject whose other poses are in the training set. This comparison highlights that our method generalizes better than NN also for shapes that should be well described by the training set. This allows us to state that navigation driven by the eigenvalues is more accurate than NN navigation of the latent space.

9 Spectral shape exploration

In the main manuscript, we show two different sub-tasks of shape exploration: shape interpolation and interactive spectrum-driven exploration.

As we claim in the main manuscript, an alternative for shape interpolation is to regard the eigenvalues themselves as a space that we can explore, as is typically done with latent spaces. In Fig. 7, on the same four low-resolution shapes, we compare interpolation in the latent space (on the right) with interpolation in the space of the eigenvalues (on the left). The former evaluation is also included in the main manuscript. For the latter, given the four shapes, we first compute their spectra, directly perform a bilinear interpolation among these spectra, and finally reconstruct the corresponding shapes. Also in this case, the entire procedure can be performed for shapes with different connectivity. The linear interpolation among spectra gives rise to meaningful results. However, observe that the space of spectra (where each point is a different sequence of eigenvalues) is not a proper vector space, and taking linear steps is not completely appropriate in this domain.

We support this with two additional figures. In Fig. 8, for four shapes in the training set, we provide a denser linear interpolation in the space of the eigenvalues. In

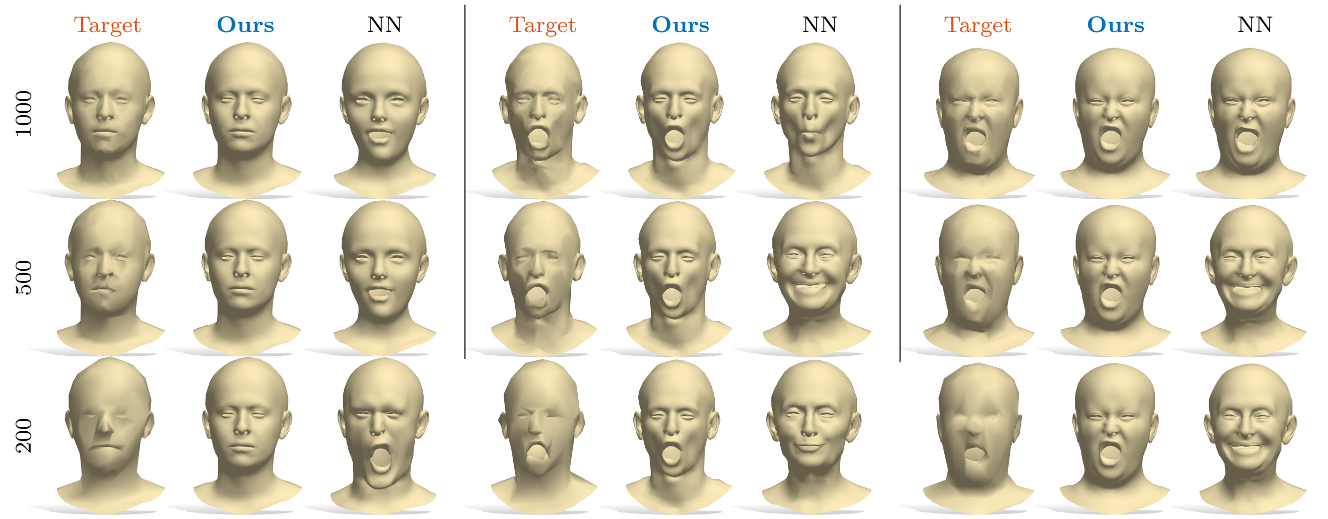

Fig. 5 Mesh super-resolution for input shapes at decreasing resolution (top to bottom, target shapes have respectively 1000, 500 and 200 vertices). Our solutions match closely with the original high-resolution version of the input shapes, while the nearest neighbor baseline (NN) predicts the wrong identity or pose.

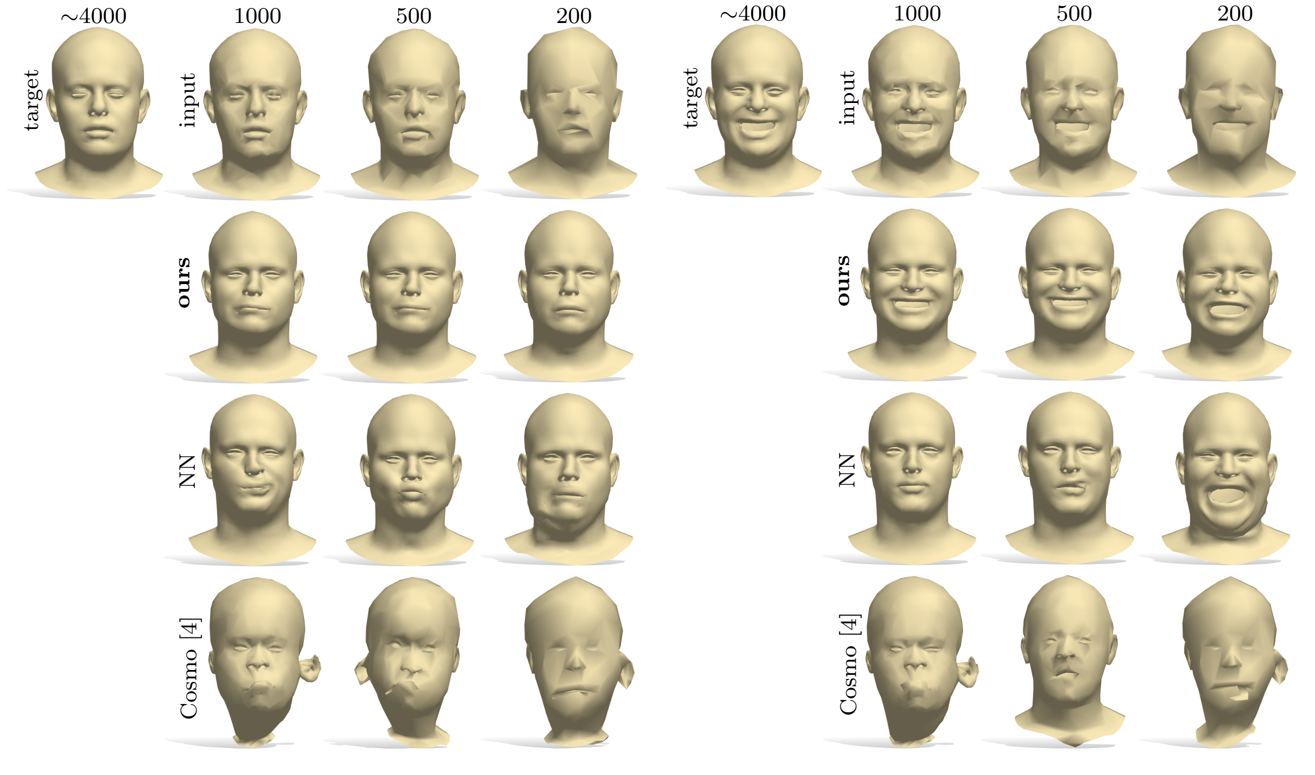

Fig. 6 Two mesh super-resolution examples for input shapes at decreasing resolution (top row of each of the two images, left to right). Our solutions match closely with the original high-resolution version of the input shapes (top left), while other approaches either predict the wrong pose (NN baseline) or generate an unrealistic shape (Cosmo *et al.*).

Fig. 9, we show the same linear interpolation between the spectra of a pair of shapes that come from two different datasets, and which were never seen in the training phase. Both cases confirm that interpolation can indeed be performed in the space of eigenvalues.

Finally, in a video attached to these supplementary materials, we show a demo of interactive spectrum-driven shape exploration. In the video, we modify the eigenvalues of a given shape and we show how these modifications allow us to interactively navigate the space of shapes.

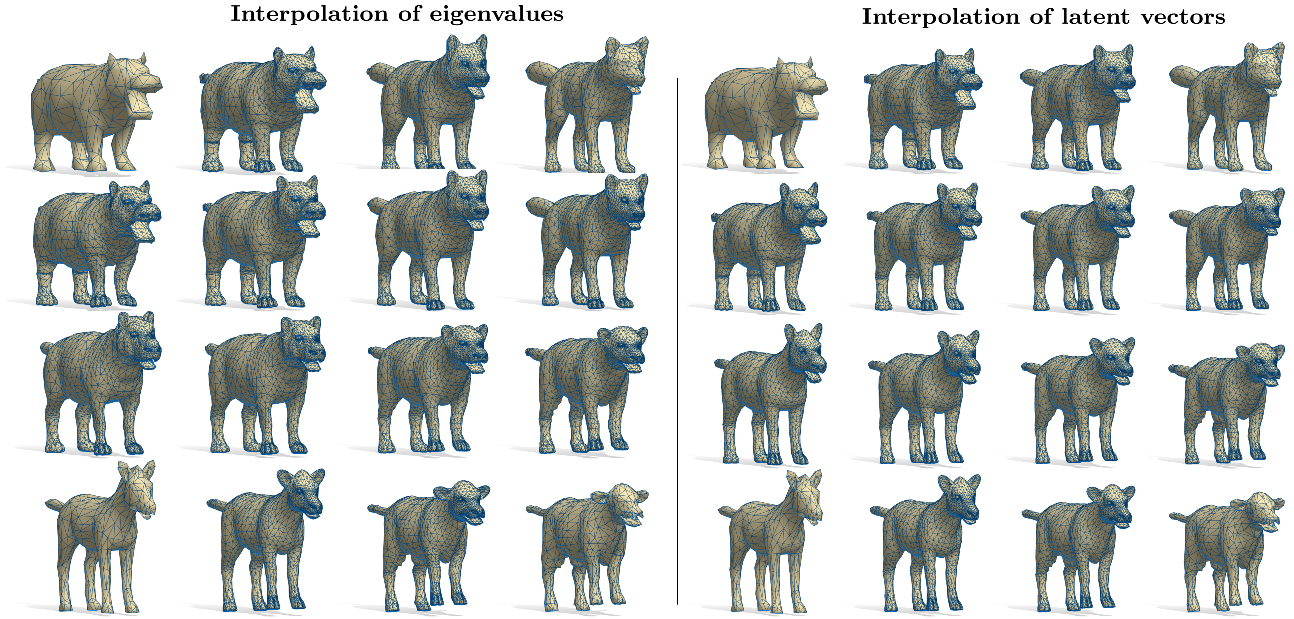

Fig. 7 Comparison between interpolation in the space of eigenvalues (left) and the interpolation in the latent space (right). The input shapes are four low-resolution versions of as many shapes from the training set.

10 Style transfer

As described in the main manuscript, we apply our trained network for the style transfer task. In Fig. 10 we show some additional examples. We emphasize here that our method is correspondence-free, since the input eigenvalues completely encode the target style. Our method also does not rely on the presence of the undeformed source shape, used in previous analogies applications, e.g. [2] that phrased the problem of finding X such that $(A \to B,\ C \to X)$ for some known A, B, C. These examples confirm the robustness and accuracy of the proposed method in this application.

References

1. Scan the world project. `https://github.com/riccardomarin/InstantRecoveryFromSpectrum`. [Online; accessed 08-February-2021]
2. Boscaini, D., Eynard, D., Kourounis, D., Bronstein, M.M.: Shape-from-operator: recovering shapes from intrinsic operators. Computer Graphics Forum **34**(2), 265–274 (2015)
3. Chang, A.X., Funkhouser, T., Guibas, L., Hanrahan, P., Huang, Q., Li, Z., Savarese, S., Savva, M., Song, S., Su, H., Xiao, J., Yi, L., Yu, F.: ShapeNet: An Information-Rich 3D Model Repository. Tech. Rep. arXiv:1512.03012 [cs.GR], Stanford University — Princeton University — Toyota Technological Institute at Chicago (2015)
4. Cosmo, L., Panine, M., Rampini, A., Ovsjanikov, M., Bronstein, M.M., Rodolà, E.: Isospectralization, or how to hear shape, style, and correspondence. In: Proceedings of the IEEE Conference on Computer Vision and Pattern Recognition (CVPR), pp. 7529–7538 (2019)
5. Huang, J., Su, H., Guibas, L.: Robust watertight manifold surface generation method for shapenet models. arXiv preprint arXiv:1802.01698 (2018)
6. Li, T., Bolkart, T., Black, M.J., Li, H., Romero, J.: Learning a model of facial shape and expression from 4D scans. ACM Transactions on Graphics, (Proc. SIGGRAPH Asia) **36**(6) (2017)
7. Qi, C.R., Su, H., Mo, K., Guibas, L.J.: Pointnet: Deep learning on point sets for 3d classification and segmentation. In: Proceedings of the IEEE Conference on Computer Vision and Pattern Recognition, pp. 652–660 (2017)
8. Ranjan, A., Bolkart, T., Sanyal, S., Black, M.J.: Generating 3D faces using convolutional mesh autoencoders. In: European Conference on Computer Vision (ECCV) (2018)
9. Reuter, M.: Hierarchical shape segmentation and registration via topological features of laplace-beltrami eigenfunctions. International Journal of Computer Vision **89**(2-3), 287–308 (2010)
10. Zuffi, S., Kanazawa, A., Jacobs, D., Black, M.J.: 3D menagerie: Modeling the 3D shape and pose of animals. In: IEEE Conf. on Computer Vision and Pattern Recognition (CVPR) (2017)

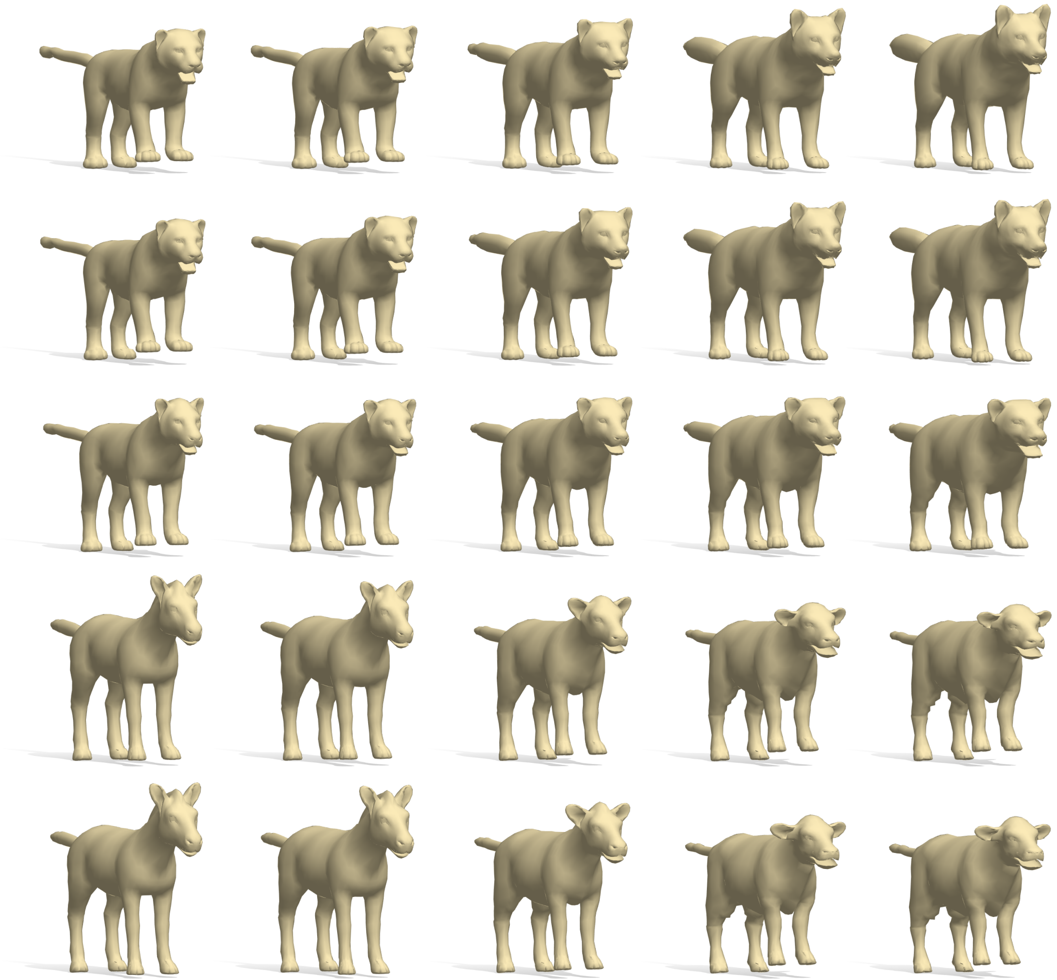

Fig. 8 Interpolation *between spectra* (as opposed to latent vectors) between four shapes in the training set.

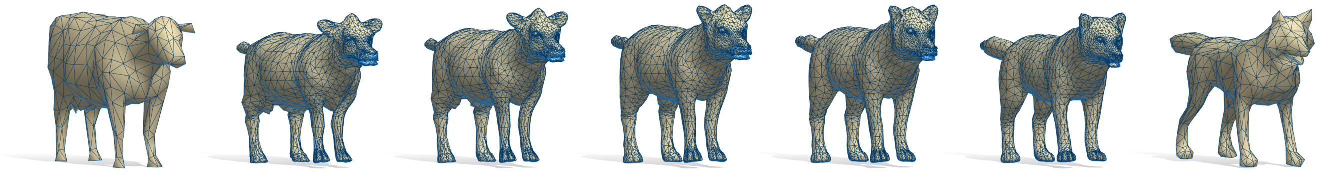

Fig. 9 Interpolation between the spectra of two low-poly shapes that belong to two different datasets, never seen at training time.

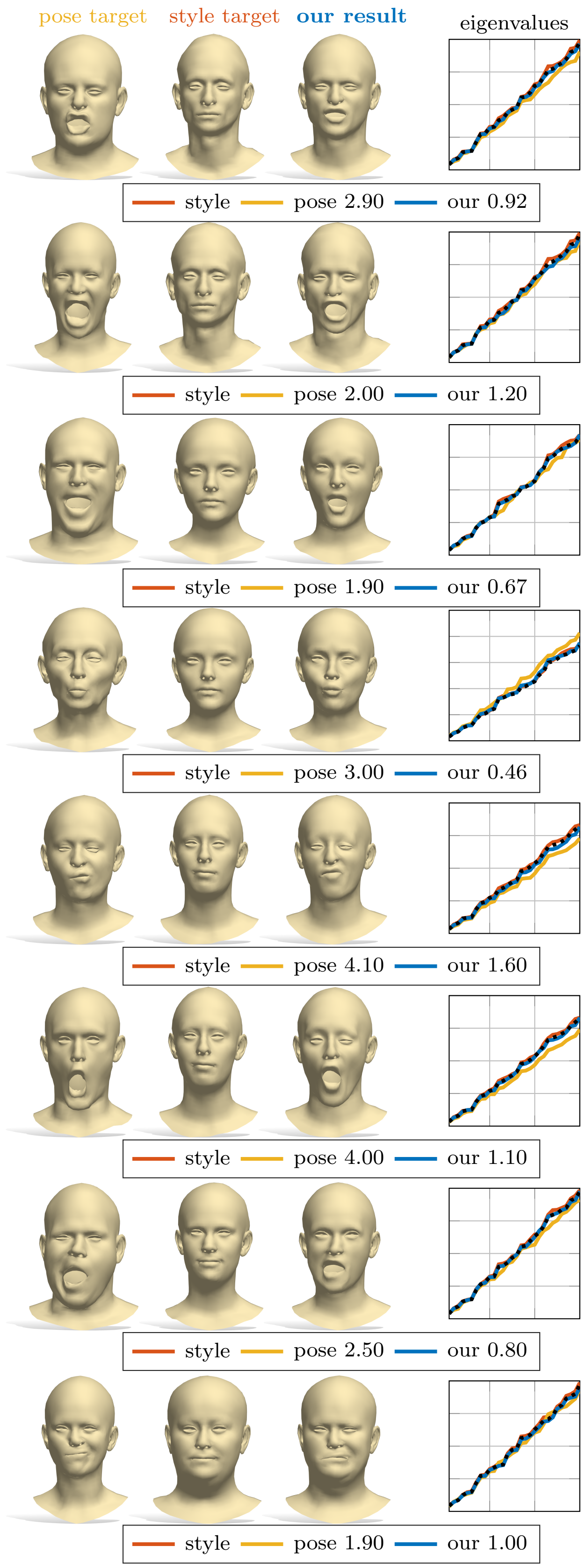

Fig. 10 Examples of style transfer. The target style (middle) is applied to the target pose (left), obtaining our result (right).
